# Encapsulation of the novel mPGES-1 inhibitor TG554 in acetalated dextran nanoparticles

**DOI:** 10.1039/d6ra01745b

**Published:** 2026-05-19

**Authors:** Franziska Adermann, Jana Ismail, Thorben Köhler, Lea C. Klepsch, Bärbel Beringer-Siemers, Carolin Kellner, Steffi Stumpf, David Pretzel, Lisa Jäpel, Ivo Nischang, Zehra Tuğçe Gür Maz, Philipp Dahlke, Hannes Engelbrecht, Paul M. Jordan, Antje Vollrath, Stephanie Schubert, Erden Banoglu, Oliver Werz, Ulrich S. Schubert

**Affiliations:** a Laboratory of Organic and Macromolecular Chemistry (IOMC), Friedrich Schiller University Jena Humboldtstraße 10 07743 Jena Germany ulrich.schubert@uni-jena.de; b Jena Center for Soft Matter (JCSM), Friedrich Schiller University Jena Philosophenweg 7 07743 Jena Germany; c Helmholtz-Zentrum Berlin für Materialien und Energie GmbH (HZB) Hahn-Meitner-Platz 1 14109 Berlin Germany; d Helmholtz Institute for Polymers in Energy Applications Jena (HIPOLE Jena) Lessingstraße 12-14 07743 Jena Germany; e Department of Pharmaceutical Chemistry, Faculty of Pharmacy, Gazi University 06560 Ankara Turkey; f Department of Pharmaceutical/Medicinal Chemistry, Institute of Pharmacy, Friedrich Schiller University Jena Philosophenweg 14 07743 Jena Germany

## Abstract

Chronic inflammatory conditions remain a major therapeutic challenge, driving the demand for more selective and well-tolerated treatment options. The 2-amino-1,3,4-oxadiazole-based TG554, a novel and highly potent inhibitor of microsomal prostaglandin E_2_ synthase-1 (mPGES-1), offers a promising route to more precisely modulate inflammation. However, its lipophilicity necessitates an advanced drug delivery approach. To overcome its solubility-related limitations, a biodegradable acetalated dextran (AcDex) nanoparticle system was developed using a robust batch nanoprecipitation protocol performed under cleanroom conditions following a predefined batch record. The resulting TG554-loaded AcDex nanoparticles exhibited favorable physicochemical properties, including a narrow size distribution profile, sufficient drug loading, and excellent colloidal stability. Their hydrodynamic properties, morphology, degradation behavior, and encapsulation efficiency were assessed using dynamic light scattering (DLS), scanning electron microscopy (SEM), and high-performance liquid chromatography (HPLC). In human pro-inflammatory M1 monocyte-derived macrophages (M1-MDM), the nanoparticles demonstrated high biocompatibility, cellular uptake, and efficient intracellular drug delivery, resulting in marked biological activity as confirmed by flow cytometry, confocal microscopy, and ultra-performance liquid chromatography tandem mass spectrometry (UPLC-MS/MS) analysis. Together, this work reports a robust biopolymer-based nanoformulation of TG554 that provides a strong foundation for further preclinical investigations and future *in vivo* evaluation.

## Abstract 2

Optimized formulation of AcDex nanoparticles for the successful delivery of the novel anti-inflammatory drug TG554 to M1 macrophages.

## Graphical abstract

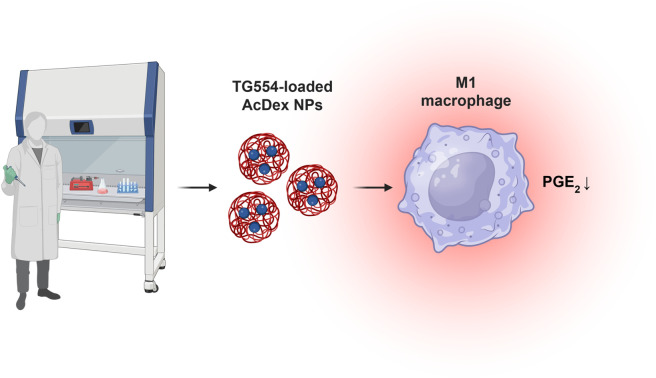

## Introduction

Inflammation is a complex physiological response crucial for host defense and tissue homeostasis. However, its chronic dysregulation plays a pathogenic role in numerous diseases, including atherosclerosis, type 2 diabetes, neurodegenerative disorders such as multiple sclerosis, and cancer.^1^ Current anti-inflammatory therapies, such as non-steroidal anti-inflammatory drugs (NSAIDs), mainly target cyclooxygenase (COX) enzymes. Concurrently, their broad inhibition of prostaglandin biosynthesis results in substantial adverse drug reactions, with gastrointestinal and cardiovascular complications being notable examples.^2^ This highlights the need for the development of novel, innovative therapeutic strategies that exhibit improved efficacy and safety profiles.

Microsomal prostaglandin E_2_ synthase-1 (mPGES-1) has emerged as a promising alternative target for the development of anti-inflammatory drugs. The inducible mPGES-1 specifically catalyzes the terminal step in the biosynthesis of prostaglandin E_2_ (PGE_2_), a key pro-inflammatory mediator, and in contrast to COX enzymes, the inhibition of mPEGES-1 does not suppress the formation of other homeostatic prostanoids.^3,4^ A more comprehensive anti-inflammatory approach is the dual inhibition of mPGES-1 and 5-lipoxygenase-activating protein (FLAP) which is involved in leukotriene biosynthesis.^5^

TG554, a novel 1,3,4-oxadiazole derivative, was identified as a highly potent and selective mPGES-1 inhibitor, exhibiting additionally suppressive effects on leukotriene biosynthesis in human macrophages.^5^ A substantial proportion of active pharmaceutical ingredients (APIs) in current development pipelines, estimated to be up to 90%, are poorly water-soluble based on the criteria of the Biopharmaceutical Classification System (BCS).^6,7^ Limited aqueous solubility adversely affects dissolution and absorption processes, resulting in low or variable bioavailability and ultimately unpredictable clinical responses. TG554 falls within this class of highly lipophilic, poorly water-soluble molecules and, therefore, faces similar challenges regarding systemic delivery and pharmacokinetic performance.^5^ To address these limitations and to enable effective systemic delivery, a suitable carrier system is required. Consequently, the main objective of this work was to develop a polymeric nanoformulation for TG554 to overcome its solubility-related constraints.

Nanoparticle-based drug delivery systems offer a versatile platform to overcome such limitations, particularly for lipophilic small molecules. These systems can solubilize hydrophobic drugs and thereby enhance bioavailability, improve pharmacokinetic profiles, and increase physicochemical stability.^8–11^ Beyond improving solubility, nanoparticle-based approaches also enable targeted drug delivery to specific cell types or tissues, which can enhance therapeutic efficacy, reduce off-target toxicity, and improve patient compliance.^12^ The first polymeric nanoparticles for drug delivery were described by Speiser *et al.*, who introduced non-biodegradable epoxy resin nanoparticles for pharmaceutical and vaccine applications.^13^ Since then, biodegradable polymers have become the predominant materials for nanoparticle fabrication. Among these, the U.S. Food and Drug Administration (FDA) approved poly(lactic-*co*-glycolic acid) (PLGA), a copolymer of poly(lactic acid) (PLA) and poly(glycolic acid) (PGA), that is widely regarded as the gold standard due to its excellent biocompatibility and predictable degradation under physiological conditions.^12,14^ Beyond PLGA, other biodegradable polyesters, such as poly(caprolactone) (PCL), poly(ester amides) (PEAs), and PLA, are also employed, alongside naturally derived polymers, including albumin, chitosan, and dextran.^15^ Among polysaccharide-based delivery platforms, dextran is particularly attractive due to its chemically neutral backbone and versatile derivatization chemistry, enabling predictable modification and good processability in nanoparticle formulations.^16,17^ A particularly promising biodegradable dextran derivative is acetalated dextran (AcDex), a pH-labile, hydrophobic derivative of the FDA-approved polysaccharide dextran first introduced by Bachelder *et al.* in 2008.^18^ AcDex exhibits excellent biocompatibility, rapid biodegradability into glucose-derived species, and broadly tunable degradation properties, rendering it a highly attractive material for drug delivery applications.^16,19^ The acid-labile acetal bonds of AcDex enable pH-responsive drug release, which is particularly advantageous when targeting sites of inflammation or intracellular compartments such as endolysosomes.^20,21^ Moreover, the release kinetics of AcDex can easily be tuned by varying the amount and ratio of cyclic to acyclic acetal functionalities *via* the reaction time.^21^

Previous studies have successfully demonstrated the encapsulation of novel hydrophobic anti-inflammatory agents, such as the dual FLAP/mPGES-1 inhibitors BRP-187 and BRP-201, in AcDex nanoparticles *via* nanoprecipitation, yielding enhanced cellular bioactivity and overcoming solubility issues.^22,23^ They even demonstrated superior activity in comparison to PLGA particles in whole blood experiments with BRP-201.^24^ Based on this knowledge, we selected AcDex as the carrier polymer for TG554 and developed a suitable batch nanoprecipitation formulation protocol. The preparation of particles *via* nanoprecipitation was first described by Fessi *et al.* in 1989 where particles are formed by interfacial deposition following solvent displacement.^25^ Since then, the method has been widely used to produce particles encapsulating hydrophobic drugs.^26^

In this study, AcDex nanoparticles encapsulating the novel mPGES-1 inhibitor TG554 were formulated using nanoprecipitation under controlled conditions aligned with good manufacturing practice (GMP) principles. The goal was to establish a robust formulation protocol (batch record) that ensures high product quality and repeatability for TG554-loaded nanoparticles. The physicochemical properties, including stability as well as the degradation profile of the TG554-loaded AcDex nanoparticles, were investigated *via* dynamic light scattering (DLS), scanning electron microscopy (SEM), and high-performance liquid chromatography (HPLC). Furthermore, their biocompatibility, biological efficacy, and cellular internalization were studied in human derived pro-inflammatory M1 monocyte-derived macrophages (MDM) using cell viability assays, flow cytometry, confocal microscopy, and ultra-performance liquid chromatography tandem mass spectrometry (UPLC-MS/MS).

## Materials

Cell adherence was performed in cell culture flasks from Greiner Bio-One (Germany) while using RPMI 1640 medium (Sigma-Aldrich, Germany). CELLview cell culture dishes, polystyrene (PS), 35/10 mm, glass bottom were purchased from the same supplier. PrestoBlue™ Cell Viability Reagent and Hoechst 33 342 were obtained from Thermo Fisher Scientific, Germany.

HPLC grade solvents (water, formic acid, acetonitrile, and dimethyl sulfoxide) were purchased from VWR, Germany. The surfactant poly(vinyl alcohol) (PVA, Parteck® MXP PVA 4-88) and the dimethyl sulfoxide (DMSO, anhydrous ≥99.9%) were purchased from Sigma-Aldrich, Germany. Acetone (99+ %, extra pure) was ordered from Thermo Scientific, Germany and triethylamine (TEA, 99%) was purchased from Thermo Scientific, Germany.

TG554 was synthesized based on established procedures.^5^ The synthesis of AcDex was based on an established procedure and is described alongside its characterization in the SI (Fig. S1–S4).^21^

## Methods

### Nanoparticle formulation

Nanoparticles were produced by nanoprecipitation in a cleanroom according to a batch record protocol that is reported in detail in the SI. All materials and equipment entering the cleanroom were sanitized according to standard operating procedures (SOPs) to minimize contamination. Shortly, the organic phase was prepared by dissolving acetalated dextran (AcDex) in acetone. The drug TG554 and the fluorescent dye neutral lipid orange (NLO) were dissolved in DMSO. For drug-loaded nanoparticles, TG554 was added to the organic phase at a drug-to-polymer ratio of 3% (w/w). For cellular uptake studies, a separate batch was prepared by adding NLO at 0.07% (w/w) relative to the polymer. The aqueous phase consisted of sterile water containing poly(vinyl alcohol) (PVA, 0.3% (w/v)) and triethylamine (TEA, 0.01% (w/v)), both added from sterile-filtered stock solutions. The organic phase was injected into the aqueous phase using a syringe pump at 2 mL min^−1^ under continuous magnetic stirring. The resulting particle dispersion was stirred at room temperature for 20 h to enable complete solvent evaporation. The nanoparticles were purified by centrifugation at 11 000 rpm for 60 min at 20 °C. The supernatant was removed, and the pellet was resuspended in sterile water containing 0.01% (w/v) TEA to a final concentration of 5 mg mL^−1^. After overnight equilibration at 4 °C, aliquots were lyophilized for subsequent analysis. Remaining suspensions were stored at 4 °C.

### Dynamic light scattering (DLS) and electrophoretic light scattering (ELS)

Nanoparticles were characterized after formulation in terms of size and polydispersity by dynamic light scattering (DLS) using a Zetasizer Ultra (Malvern Panalytical, UK) with a laser wavelength of *λ* = 633 nm. Measurements were performed after solvent evaporation, post-centrifugation, and after resuspension in water with 24 h equilibrium time in between using polystyrene UV cuvettes (Brand) at 25 °C. Intensity fluctuations at a backscattering angle of 173° were measured to determine the particle size in the form of z-average hydrodynamic diameter (*d*_h_) and polydispersity index (PDI) values with 30 s equilibration time and five runs of 30 s acquisition time. For the measurements, 10 µL of the particle dispersions were diluted to 100 µL with ultrapure water. Three independent batches were measured. The particles' electrophoretic potential, also called zeta potential (*ζ* in mV), was measured three times at 25 °C in automatic mode. For this purpose, 10 µL of the particle dispersion were diluted to 1 mL with water and filled into a folded capillary zeta cell (Malvern Panalytical, UK). The data is reported as average and standard deviation among all repeated formulations.

### Scanning electron microscopy (SEM)

The blank and TG554-loaded AcDex particles were pipetted onto mica substrates and air dried for 1 h. Subsequently, the dried nanoparticles on the substrate were coated with a thin layer of platinum (4 nm) using sputter coating (CCU-010 HV, Safematic GmbH, Switzerland).^27^ Afterwards, the samples were imaged with a Sigma VP field emission scanning electron microscope (Carl-Zeiss AG, Germany) that acquired the images with an InLens detector using an acceleration voltage of 4 kV and 6 kV.

### High-performance liquid chromatography (HPLC)

The lyophilized nanoparticles were dissolved in a mixture of DMSO/CH_3_CN 50/50 (%, v/v) to a final concentration of 1 mg mL^−1^ using 5 min of ultrasonication at room temperature. To establish a calibration curve for TG554, a concentration series of 5, 10, 15, 20, 25, and 50 mg mL^−1^ of the drug was prepared. Before chromatographic analysis, all solutions were filtered through a 0.45 µm polytetrafluoroethylene (PTFE) filter. Chromatographic analysis was performed on an UltiMate™ 3000 Rapid Separation (RS) Ultra High-Performance Liquid Chromatography (UHPLC) system from Thermo Fisher Scientific, USA. A monolithic Chromolith® High Resolution RP-18 endcapped column (100 × 4.6 mm) from Merck KGaA was used as the stationary phase. The autosampler temperature was set to 17 °C and the column oven temperature was set to 35 °C. The injection volume was 5 µL and a flow rate of 1 mL min^−1^ was utilized for chromatographic elution. For detection, a diode array detector (DAD), operating at a wavelength of 287 nm was used. Data were acquired at an acquisition frequency of 5 Hz. The binary mobile phase solvent composition consisted of CH_3_CN and 0.1% formic acid (FA) in water (v/v). For drug elution, the binary mobile phase solvent composition was held constant at 70/30 CH_3_CN/0.1% aqueous FA (%, v/v) for 5 min, followed by a linear gradient to 100% CH_3_CN within 2 min. After holding constant the mobile phase composition for 6 min, the CH_3_CN content was linearly decreased to the initial 70% within 1 min. The column was subsequently equilibrated for 3 min before the next injection. Data evaluation was performed using the Thermo Scientific Dionex Chromeleon 7 Chromatography Data System software.

### Isolation of monocytes and generation of proinflammatory monocyte-derived macrophages

Monocyte isolation and generation of proinflammatory monocyte-derived macrophages was performed based on a previously described method.^28^ Leukocyte concentrates derived from freshly withdrawn blood (16 I.E. heparin mL^−1^ blood) of healthy adult male and female volunteers (18 to 65 years, without details about ancestry, race, or ethnicity) were provided by the Department of Transfusion Medicine at the University Hospital of Jena, Germany. The experimental procedures were approved by the local ethical committee (approval no. 5050-01/17) and were performed in accordance with the guidelines and regulations. Voluntary donors agreed to the usage *via* written consent. Following an established protocol,^29^ peripheral blood mononuclear cells (PBMC) were isolated using density gradient centrifugation after the addition of a lymphocyte separation medium (Histopaque®-1077, Sigma-Aldrich, Germany) and after the sedimentation of erythrocytes by dextran. PBMC were seeded in cell culture flasks (Greiner Bio-one) in PBS pH 7.4 with CaCl_2_ and MgCl_2_ (Sigma-Aldrich). After 1 h at 37 °C and 5% CO_2_ for adherence of the monocytes, the medium was discarded and replaced with RPMI 1640 (Sigma-Aldrich) containing 10% (v/v) heat-inactivated fetal calf serum (FCS), 2 mmol L^−1^ glutamine (Biochrom/Merck, Germany), 100 U penicillin, and 100 µg mL^−1^ streptomycin (Biochrom/Merck, Germany). For differentiation towards macrophages, the monocytes were kept in RPMI 1640 supplemented with 10% (v/v) FCS, 2 mmol L^−1^ glutamine, 100 U mL^−1^ penicillin, and 100 µg mL^−1^ streptomycin for six days with 20 ng mL^−1^ GM-CSF (PeproTech, USA) for M0 differentiation. Afterwards, M0 were incubated with 100 ng mL^−1^ LPS (lipopolysaccharide) and 20 ng mL^−1^ IFN-γ (PeproTech, USA) for another 24 h to obtain M1-MDM.

### Incubation of M1-MDM

After polarization to M1-MDM, the medium was discarded, and phosphate buffered saline (PBS) plus 1 mM calcium chloride (CaCl_2_) was added to the cells. After preincubation for 30 min with the vehicle (PBS), TG554 encapsulated in nanoparticles (AcDex[TG554]; 1 µM final TG554 concentration) or blank nanoparticles (AcDex[Blank]), the cells were stimulated with 1% *Staphylococcus aureus*-conditioned medium (SACM) for 90 min at 37 °C and 5% CO_2_. SACM was prepared as previously described.^30^ Incubation was stopped by adding methanol, and lipid mediators (LMs) were analyzed as described by LM metabololipidomics below.

### LM metabololipidomics by UPLC-MS/MS

After the incubation, the supernatants were transferred to 2 mL of ice-cold methanol containing 10 µL of deuterium-labelled internal standards (200 nM d8-5S-HETE, d4-LTB_4_, d5-LXA_4_, d5-RvD_2_, d4-PGE_2_, and 10 µM d8-AA; Cayman Chemical/BiomolGmbH, Germany) to facilitate quantification and sample recovery. Sample purification was accomplished as published recently.^31^ In brief, samples were kept at −20 °C for at least 60 min to allow protein precipitation. Centrifugation (1200×*g*, 4 °C, 10 min) and acidification using 9 mL acidified water (final pH 3.5) were followed by solid phase extraction (SPE). Solid phase cartridges (Sep-Pak®Vac 6cc 500 mg/6 mL C18; Waters, USA) were washed with 6 mL of methanol and 2 mL of water before the samples were loaded onto columns. After washing with 6 mL of water and additional 6 mL of *n*-hexane, LM were eluted with 6 mL of methyl formate. Finally, the samples were evaporated using an evaporation system (TurboVap LV, Biotage, Sweden) and resuspended in 100 µL of a methanol–water mixture (50/50, v/v) for UPLC-MS/MS automated injections. For UPLC-MS/MS, an Acquity UPLC system (Waters, USA) and a QTrap 5500 Mass Spectrometer (Sciex, USA) equipped with an electrospray ionization source were employed, as described before.^31^ The identity of low abundance analytes was confirmed by fragmentation pattern matching by re-analysis using a QTrap 7500 mass spectrometer (Sciex, USA) controlled by SCIEX-OS and comparing the enhanced product ion scans of the biological sample with that of authentic standards.

### Investigation of the cellular toxicity of the nanoparticles

To evaluate the safety profile of the prepared particles, PrestoBlue™ assay was performed as an *in vitro* cell viability assay, following the manufacturer's instructions. Unpolarized human monocyte-derived macrophages (M0-MDM) were seeded into 96-well plates at a density of 2 × 10^5^ cells per well in RPMI 1640 media supplemented with FCS, l-glutamine, penicillin and streptomycin (P/S), LPS and IFN-γ, as described above, and left to polarize to M1-MDM over 24 h. Subsequently, the cells were incubated with three different concentrations of nanoparticles (1.64 µg mL^−1^ [0.1 µM TG554], 16.43 µg mL^−1^ [1 µM TG554], and 164.3 µg mL^−1^ [10 µM TG554]) at 37 °C and 5% CO_2_ for 24 h (three experimental replicates (three donors), three technical replicates, one formulation batch). Sample dilutions were prepared beforehand in ultrapure water (16.43 µg mL^−1^, 164.3 µg mL^−1^, and 1643 µg mL^−1^). Then, by directly applying the sample pre-dilutions into the media of the corresponding wells (1 : 10), the target sample concentrations were reached, and disturbance of cell integrity by media exchange was avoided. As controls, MDM were incubated with fresh media supplemented with 10% (v/v) ultrapure water instead of nanoparticles. After 24 h, the media were replaced with 100 µL of fresh media containing 10% (v/v) PrestoBlue™. After incubation for 45 min at 37 °C, fluorescence was measured at *λ*_ex_ = 560 nm and *λ*_em_ = 590 nm in top reading mode using the Infinite M200 Pro plate reader (Tecan Group). The percentage of cell viability as compared to the untreated cells is being reported as cytotoxicity, and as per DIN EN ISO 10993-5, values below 70% viability are considered toxic.

### Investigation of nanoparticle internalization *via* flow cytometry

To evaluate the internalization potential of the nanoparticles into M1-MDM as well as to assess whether the encapsulated drug could affect uptake, AcDex particles were prepared encapsulating the dye NLO either with or without TG554 (AcDex[TG554/NLO] or AcDex[NLO]). A similar MDM polarization setup as previously described in the cytotoxicity section was followed, where M0-MDM were plated into 96 well plates at a density of 2 × 10^5^ cells per well and left to polarize to M1-MDM over 24 h. Following that, the MDM were treated with the nanoparticles at a concentration of 16.43 µg mL^−1^ (1 µM of TG554) for 30 min. As controls, MDM were incubated with fresh media supplemented with 10% (v/v) ultrapure water instead of particle samples. Then, the medium was discarded, and cells were washed once gently with PBS, detached with PBS containing 5 mmol L^−1^ EDTA and left in the same plate. MDM were then analyzed *via* flow cytometry using the CytoFlex LX (Beckman Coulter GmbH, Germany).

To exclude debris, other cells as well as aggregates, and to set the proper gate for MDM, the side scatter and forward scatter signals were employed. Particle internalization was analyzed in 6500 events based on NLO fluorescence which was measured using an excitation wavelength of 561 nm and a 585/42 bandpass filter. Data analysis was performed using the CytExpert 2.5 software (three experimental replicates (three donors), three technical replicates, one formulation batch). The mean fluorescence intensity (MFI) values were adjusted using a correction factor derived from the fluorescence measurement of the particle dispersions.

### Investigation of nanoparticle internalization *via* confocal laser scanning microscopy (CLSM)

To further assert the particle internalization and to confirm the intracellular localization of the nanoparticles (*vs.* cell surface attachment) as well as to study the time dependency of the uptake, CLSM was employed. M0-MDM were seeded into CELLview cell culture dishes (PS, 35/10 mm, glass bottom, four compartments) at a density of 5 × 10^5^ cells per well (two experimental replicates) and left to polarize to M1-MDM over 24 h. Subsequently, the cells were stained with 10 µg mL^−1^ Hoechst 33 342 (Invitrogen, USA) for 10 min and treated with 16.43 µg mL^−1^ (1 µM TG554) of particles. While imaging, the confocal dishes were placed in a CLSM incubation system maintained at 37 °C in a humidified atmosphere containing 5% CO_2_. MDM were imaged at 30 min, 45 min, and 60 min using Zeiss LSM 880 Elyra (Carl Zeiss, Germany). Hoechst 33 342 and NLO channels were excited at 405 nm and 561 nm, respectively. Images from an ROI of 236 × 236 µm were acquired using a 40× water-immersion objective (N.A. 1.2) with a pixel size of 160 nm.

## Results and discussion

### Nanoparticle formulation and characterization

**Fig. 1:**
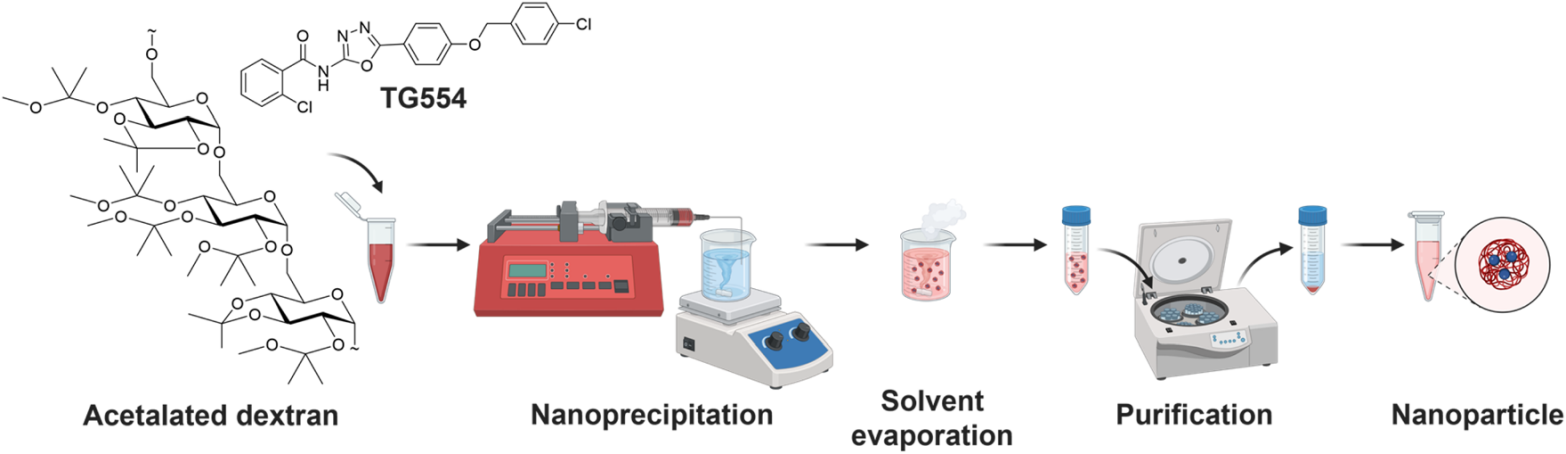
Schematic representation of the batch nanoprecipitation of AcDex nanoparticles encapsulating TG554.

A reliable formulation protocol using batch nanoprecipitation was developed from previously successfully established AcDex formulations with anti-inflammatory drugs (Fig. 1).^22,24^ Herein, an initial polymer concentration of 10 mg mL^−1^ and acetone as the organic phase to dissolve the polymer AcDex was used. Acetone can be easily removed by solvent evaporation at ambient temperature, and it is classified as class 3 solvent with low toxic potential, as stated by the International Council for Harmonisation of Technical Requirements for Pharmaceuticals for Human Use (ICH) guideline for residual solvents.^32^ To dissolve the lipophilic drug TG554, DMSO, also a class 3 solvent, was used as acetone was unable to dissolve it sufficiently.^32^ Although literature indicates that AcDex enables the encapsulation of high amounts of hydrophobic drugs (≥10% loading),^24^ the initial drug feed was set to 3% (w/w related to polymer) to circumvent possible drug precipitation and to ensure full drug encapsulation. TG554 is a highly active drug, and pure drug precipitates may interfere with bioactivity studies and would lead to the need for elaborate purification methods. Partially hydrolyzed PVA was utilized as a surfactant to stabilize the particles during the formulation and the purification processes. The pH value of the aqueous solution was adjusted to slightly basic conditions (pH > 7.5) using TEA. Particles were produced in triplicates and subsequently analyzed. The corresponding batch records and detailed characterization data can be found in the SI Tables S1–S8.

**Fig. 2:**
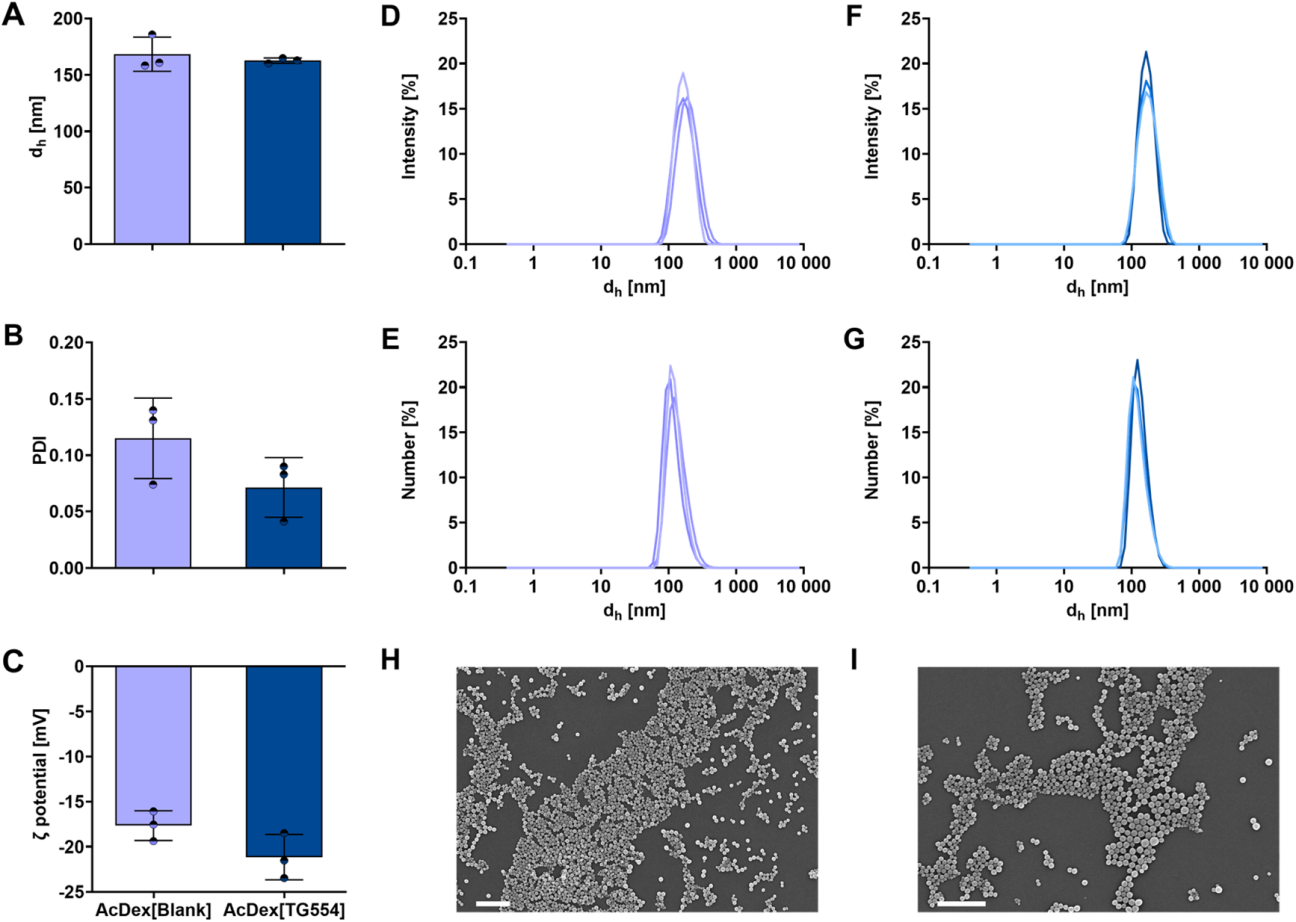
(A) Nanoparticle sizes (*z*-average value represented as *d*_h_) and (B) polydispersity index (PDI) values obtained by dynamic light scattering. (C) Zeta potential (*ζ*) obtained by electrophoretic light scattering. (D) Intensity and (E) number distribution of particle size of AcDex[Blank] nanoparticles. (F) Intensity and (G) number distribution of particle size of AcDex[TG554] nanoparticles. Data is presented as mean values ± standard deviation (SD). *n* = 3 for different batches of manufactured nanoparticles. Scanning electron microscopy images of AcDex[Blank] (H) and AcDex[TG554] (I) nanoparticles (scale bar = 1 µM).

The physicochemical properties of the AcDex nanoparticles were investigated following the formulation by nanoprecipitation and solvent evaporation, as well as after the purification by centrifugation. DLS analysis revealed that both the AcDex[Blank] and the AcDex[TG554] nanoparticles exhibited an apparent size below 200 nm (Fig. 2(A), Tables S7, and S8). This size range has previously been discussed in the context of macrophage uptake of nanoparticles.^33–35^ Through both the blank and the drug-loaded formulations, the polydispersity index (PDI) values remained below 0.15. Given that PDI values at or below 0.2 are commonly considered to be acceptable for polymeric nanoparticles,^36^ the low PDI observed here indicates a narrow and well-controlled particle size distribution.

Low PDI values are essential for the repeatability and scalability of formulations as well as for ensuring consistent physicochemical and biological behavior.^36^ As shown in Fig. 2, the incorporation of TG554 did not significantly affect the average particle size up to the used drug feed of 3% (w/w). In contrast, the width of the size distribution of AcDex[TG554], represented by PDI values, was narrower compared to the blank controls (PDI in Fig. 2(B), intensity and number distribution in Fig. 2(D–G) and S5). There appears to be a stabilizing effect of the TG554 within the polymeric core during self-assembly, likely due to the hydrophobic nature of the drug.^37^

Both blank and TG554-loaded AcDex nanoparticles exhibited comparable zeta potential values ranging from −18 to −21 mV (Fig. 2(C)). Although the absolute zeta potential is not sufficiently high to provide strong electrostatic stabilization, values in this range generally enable a short-term stability.^38^ The similarity between the two formulations indicates that the drug encapsulation does not markedly alter the surface charge characteristics of the nanoparticles.

In addition to DLS, SEM measurements were performed to gain further information about the particles' size, morphology, and the presence of drug precipitates, a known challenge for polymeric drug formulations.^39,40^ The electron microscopy images confirmed the formation of individual particles with uniform spherical morphology and agreed with the DLS data, with no apparent evidence of particle clustering or aggregation (Fig. 2(H and I)). More importantly, no separate free drug precipitates were observed ensuring sufficient encapsulation of the API.

To evaluate the encapsulation efficiency (EE) achieved by the formulation process, the drug content of the TG554-loaded nanoparticles was quantified using HPLC of dissolved AcDex[TG554] nanoparticle samples. Though the drug coeluted with the polymer as seen in initial studies with a charged aerosol detector, drug elution was not compromised as seen in elution profiles of varying stock solutions of drug and dissolved AcDex[TG554] monitored by UV detection (Fig. S6). Also, there is an absence of drug signal when eluting dissolved AcDex[Blank] utilizing DAD (Fig. S7). A high correlation coefficient for the drug calibration curve is obtained (Fig. S8). The drug loading capacity (LC) was estimated to be 2.19% (Table S8). The high encapsulation level of 73% obtained from an initial drug feed of 3% (w/w, drug/polymer) and a low variance across the formulations indicate an efficient, robust, and repeatable encapsulation process. This suggests high compatibility of the hydrophobic TG554 with AcDex, similar to other highly hydrophobic, anti-inflammatory drugs like BRP-187 or BRP-201.^22,24^

### Nanoparticle stability

**Fig. 3:**
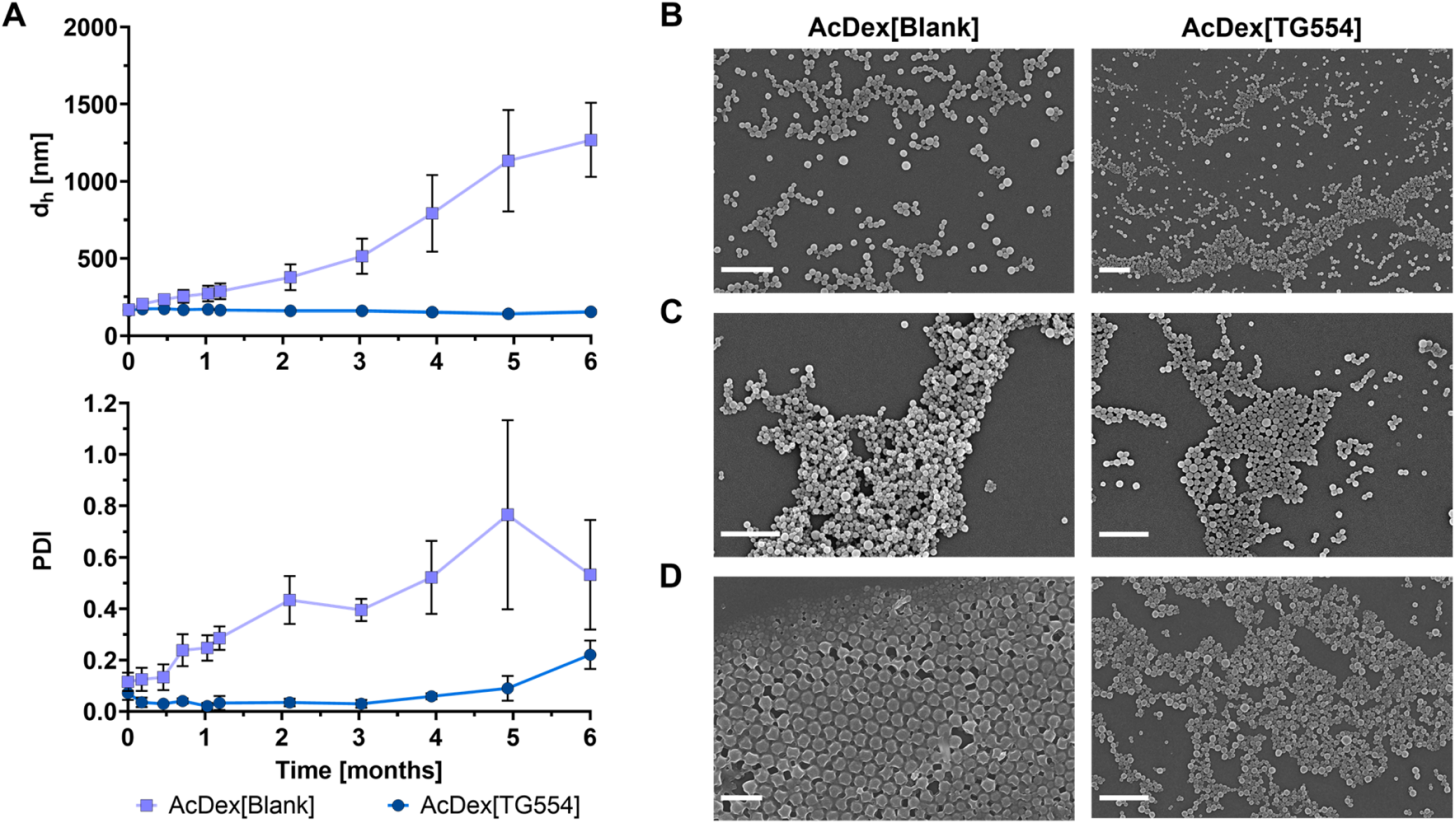
(A) Mean particle size (*z*-average value represented as *d*_h_) and polydispersity index (PDI) values obtained by dynamic light scattering after formulation (0 months) and after storage (1 to 6 months) at 4 °C. Data are presented as mean values ± SD. *n* = 3 for different batches of manufactured nanoparticles. Scanning electron microscopy images of AcDex[Blank] and AcDex[TG554] after (B) one month, (C) two and (D) four months of storage at 4 °C (scale bar = 1 µM).

Stability studies at 4 °C conducted over a six-month storage period provided insight into the long-term colloidal behavior and degradation of the formulations. SEM measurements over this period revealed no apparent degradation of the particles (Fig. 3(B–D) and S10). In contrast, AcDex[Blank] nanoparticles exhibited a pronounced increase in size and dispersity as indicated by DLS measurements, suggesting progressive aggregation over time, beginning after 20 days (Fig. 3(A) and S9). Particle aggregation represents a common challenge in nanocarrier systems, particularly those with hydrophobic cores suspended in aqueous media, where interparticle attractions are promoted to reduce the total free energy of the system.^41^ The instability observed in the blank nanoparticles is consistent with this phenomenon, confirming their susceptibility to such non-specific interactions. In contrast, the AcDex[TG554] nanoparticles maintained stable physicochemical properties throughout the same period, exhibiting only a minor increase in dispersity after six months of storage. This consistent size and PDI values suggest a high degree of long-term colloidal stability (Fig. 3). Since the zeta potential measurements of both nanoparticle types revealed comparable values (−18 to −21 mV; Fig. 2(C)), electrostatic stabilization due to the electrostatic repulsion between similarly charged particles^38^ alone does not account for the markedly improved stability of the TG554-loaded formulation. Instead, the data suggests that the encapsulation of TG554 provides an additional, central stabilizing effect on the nanoparticles. It can be hypothesized that strong drug–polymer interactions within the hydrophobic core of the AcDex particles reinforce the internal structure and reduce non-specific hydrophobic interactions between adjacent particles in suspension. The observation that the encapsulated drug itself seems to contribute to the long-term colloidal stability has been described in previous studies reporting that efficient hydrophobic drug incorporation into polymer matrices enhances intermolecular interactions and stabilizes nanocarrier assemblies,^42^ which is highly relevant for establishing an adequate shelf life and improving the overall clinical translation of the TG554 formulation.

To complement the long-term storage studies, the short-term stability of the TG554-loaded AcDex nanoparticles was evaluated under physiological and acidic conditions. Stability in PBS is essential for maintaining nanoparticle integrity during handling, administration, and early systemic circulation. In acidic environments, a rapid degradation is expected for AcDex due to its pH-labile acetal groups. This degradation profile is required to enable drug release in acidified biological compartments like endosomes or inflamed tissues.^43^ The nanoparticles were therefore incubated in water (pH 6.5), PBS (pH 7.4), and acetate buffer (pH 5) over a period of 24 h. The hydrodynamic size and dispersity were monitored over time next to the count rate using DLS. In order to investigate the behavior of the particles in their dispersant and to compare this to the behavior of the other media, water was used as a control. In PBS, both blank and TG554-loaded particles remained colloidally stable throughout the incubation period, showing only a slight increase in size (Fig. S11 and S12). The addition of PVA to PBS was able to prevent this size increase, further stabilizing the particle suspension (Fig. S14 and S15). This stability is consistent with previous reports, indicating that AcDex nanoparticles retain their structural integrity under neutral conditions.^20^ In contrast, incubation in acetate buffer resulted in a decrease in count rate as well as an increase in particle size, reflecting the expected acid-triggered degradation of AcDex (Fig. S11 and S12). This confirms that the formulation exhibits pH-responsive behavior, which is a requirement for controlled drug release from AcDex-based systems. Although direct quantification of TG554 release was not possible due to the drug's high hydrophobicity and rapid precipitation of the free drug,^44,45^ nanoparticle degradation serves as an indirect indicator that drug release is feasible under acidic conditions. Additionally, the observed biological activity of the nanoparticles, which will be discussed later, suggests the liberation of TG554 from the formulation (Fig. 5). Together, the short-term stability studies demonstrate that the TG554-loaded AcDex nanoparticles are sufficiently stable under physiological conditions, yet rapidly degrade under acidic conditions, supporting their suitability for targeted drug release in inflamed tissue.

### Biosafety and cellular uptake of AcDex[NLO] and AcDex[TG554/NLO] nanoparticles

To evaluate the safety profile and cellular uptake of the nanoparticles, blank and TG544-loaded AcDex nanoparticles were prepared with the addition of the fluorescent dye NLO that enabled an *in vitro* examination. Both particle systems, AcDex[NLO] and AcDex[TG554/NLO], were thoroughly characterized (Tables S9 and S10 as well as Fig. S13 and S16) and subsequently assessed using the PrestoBlue™ cell viability assay. A previous study by Maz *et al.* demonstrated that TG554 is non-toxic to human unpolarized monocyte-derived macrophages (MDM) at concentrations of 1 µM and 10 µM.^5^ Furthermore, the safety of AcDex-based particles has been well established in multiple studies.^18,21,46^ Therefore, this assay aimed to validate the compatibility of the drug–carrier combination with human immune cells prior to further biological evaluations. To this end, M1-MDM, as pro-inflammatory sentinels, were exposed to increasing concentrations of nanoparticles corresponding to 0.1 µM, 1 µM, and 10 µM of TG554 (nanoparticle concentrations: 1.64 µg mL^−1^, 16.43 µg mL^−1^ and 164.3 µg mL^−1^, respectively) for 24 h.

**Fig. 4:**
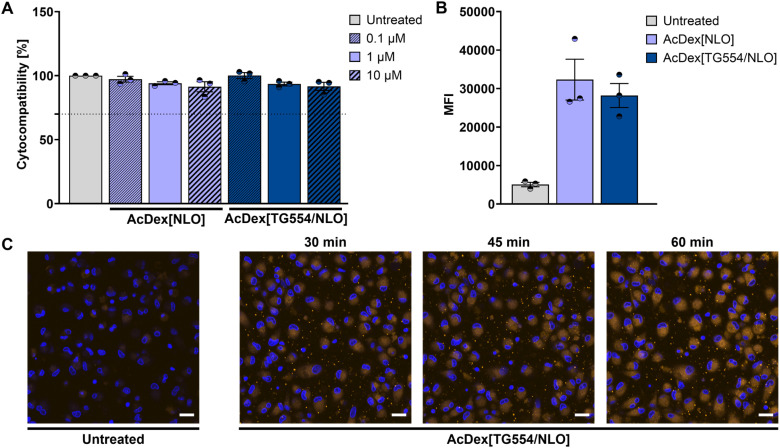
Biological investigation of biocompatibility and cellular internalization of the AcDex nanoparticles studied. All particles were loaded with the fluorescent dye NLO. (A) Cytotoxicity of AcDex[NLO] and AcDex[TG554/NLO] nanoparticles in M1-MDM at three different concentrations (corresponding to 0.1, 1, and 10 µM of TG554) at 24 h (*n* = 3 of one formulation batch). (B) Uptake of the blank and TG554-loaded AcDex nanoparticles in M1-MDM at a concentration corresponding to 1 µM of TG554 at 30 min post-treatment (*n* = 3 of one formulation batch). MFI = mean fluorescence intensity. Data are presented as mean values ± standard error of the mean (SE). (C) Confocal laser scanning microscopy imaging of AcDex particles uptake kinetics in M1-MDM at a concentration corresponding to 1 µM of TG554 (blue: Hoechst 33 342; orange: NLO) (scale bar = 20 µm).

As shown in Fig. 4(A) and Table S11, neither the blank nor the TG554-loaded nanoparticles induced any reduction in metabolic activity of M1-MDM (>70%), deeming the formulations non-toxic across the tested concentration range. Investigating cellular internalization of the nanoparticles is also critical to assess the delivery efficiency and to determine whether the presence of the encapsulated drug affects particle uptake. The encapsulation of NLO into the particles allowed efficient tracking of their uptake. As observed in Fig. 4(B) and Table S12, AcDex[NLO] and AcDex[TG554/NLO] nanoparticles were taken up efficiently by the MDM within 30 min, as quantified by flow cytometry. This rapid internalization highlights the high endocytic capacity of M1-MDM and suggests that the characteristics of AcDex-based formulations facilitate swift cellular recognition.^47,48^ No significant difference in uptake was observed between drug-loaded and blank particles, indicating that the presence of TG554 does not impair internalization. This is particularly relevant given that previous studies have shown that certain drugs, when encapsulated at high concentrations, can reduce nanoparticle uptake by altering particle surface properties, aggregation state or protein adsorption.^49,50^ This supports the suitability of TG554 for encapsulation in AcDex-based carriers.

To further investigate nanoparticle uptake kinetics, confocal microscopy was employed. As shown in Fig. 4(C), a prominent NLO fluorescence signal was visible in the cytoplasm of MDM as early as 30 min post-treatment with TG554-loaded nanoparticles. This signal increased progressively at 45 and 60 min, confirming time-dependent internalization of the nanoparticles. Importantly, cellular morphology and integrity remained unaffected throughout the experiment. Overall, these findings highlight the biocompatibility and efficient cellular uptake of the studied AcDex nanoparticles, underscoring their potential as promising delivery vehicles for TG554 and other hydrophobic therapeutics.

### Investigation of the bioactivity of AcDex[Blank] and AcDex[TG554] nanoparticles

Targeted inhibition of mPGES-1 is recognized as a potential therapeutic approach for the development of next-generation anti-inflammatory drugs. TG554 was recently shown to be a potent mPGES-1 inhibitor in a cell-free assay, with an IC_50_ of 5.6 nM. In comprehensive LM metabololipidomics using activated human M1-MDM, TG554 was further found to selectively inhibit the formation of inflammatory PGE_2_ while leaving all other COX-derived prostanoids unaffected. In the latter macrophage-based assay, much higher concentration (1 µM) was required to significantly inhibit PGE_2_ formation. Additionally, TG554 did not cause substrate shunting towards the 5-LOX pathway and did not interfere with the synthesis of specialized pro-resolving mediators, unlike traditional COX inhibitors.^5^ In the current study, to enhance the bioavailability and effectiveness of TG554 in the cellular context, the API was encapsulated in nanoparticles. Three individually formulated batches of each nanoparticle system, either TG554-loaded or blank, were tested for their ability to suppress mPGES-1 activity in human pro-inflammatory M1-MDM. The experiment was conducted at a single concentration and time point as a proof-of-concept assessment of biological activity. The primary objective was to confirm that the encapsulation of the compound as well as its cleanroom processing do not compromise its known biological activity and selectivity.

M1-MDM were preincubated with AcDex[TG554] and AcDex[Blank] nanoparticles for 30 min at a concentration of 1 µM for TG554. Subsequently, the macrophages were stimulated with 1% SACM to activate LM pathways, including 5-LOX and COX product formation, as previously described.^5,30^ The applied short preincubation time of 30 min of the nanoparticles before cell stimulation was well justified by the physicochemical and cellular behavior of the TG554-loaded nanoparticles outlined in this study. As previously described, the particles exhibited satisfactory colloidal stability at physiological pH value while being rapidly taken up by cells, ensuring efficient intracellular delivery during the applied incubation period. Upon internalization into macrophages, the acidic intracellular environment promotes a rapid degradation of the AcDex particles, resulting in TG554 release.

**Fig. 5:**
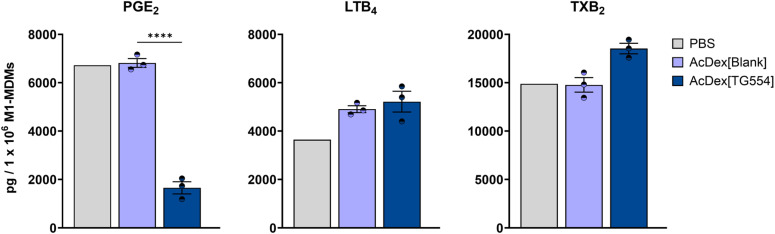
Human M1-monocytes-derived macrophages (M1-MDM, 1 × 10^6^) were preincubated with PBS, 1 µM encapsulated TG554 (AcDex[TG554]) or blank nanoparticles (AcDex[Blank]) for 30 min and subsequently stimulated with 1% *Staphylococcus aureus*-conditioned medium for 90 min at 37 °C and 5% CO_2_. Supernatants were collected, formed lipid mediators were extracted by solid phase extraction and analyzed by ultra-performance liquid chromatography tandem mass spectrometry. Results are presented as pg/1 × 10^6^ M1-MDM as mean ± SE. with single values. *n* = 3 for different batches of manufactured nanoparticles. For statistical analysis unpaired student's *t*-test was performed; ****p < 0.0001.

At a nominal concentration of 1 µM of TG554, all three AcDex[TG554] nanoparticle batches significantly reduced the formation of PGE_2_ in M1-MDM, while blank nanoparticles did not affect PGE_2_ formation (Fig. 5 and Table S13). In contrast, other bioactive LM, such as leukotriene B_4_ (LTB_4_) or thromboxane B_2_ (TXB_2_), remained unaffected by both AcDex[TG554] and AcDex[Blank] particles (Fig. 5), consistent with the results of the free drug experiments and established selectivity profile of TG554 reported by Maz *et al.*^5^ Moreover, blank-loaded nanoparticles did not trigger LM formation in M1-MDM overall, supporting their safe application without inducing cellular activation (data not shown). Collectively, this data confirms that the encapsulation of TG554 into polymeric nanoparticles retains the established biological selectivity of TG554, effectively inhibiting mPGES-1 activity in human primary M1-MDM.

## Conclusion

Acetalated dextran nanoparticles were successfully formulated using a nanoprecipitation protocol specifically optimized for the hydrophobic mPGES-1 inhibitor TG554. The resulting nanoparticles displayed a uniform size of approximately 163 nm with a PDI of 0.07 and a drug loading of 2.19% as determined by HPLC. Long-term storage studies indicate colloidal stability for a period of up to six months at 4 °C, suggesting that TG554 functions as a potent internal stabilizer. Furthermore, the particles displayed sufficient stability in PBS and a rapid pH-triggered degradation in acetate buffer, consistent with the acid-labile nature of AcDex. This pH-responsiveness is particularly advantageous for targeting inflamed tissues, which often exhibit lower extracellular pH value, and acidic endolysosomal compartments, thereby enabling environment-specific drug release. In human pro-inflammatory M1-MDM, the nanoparticles were rapidly internalized, as demonstrated by flow cytometry and confocal microscopy, and revealed no cytotoxic effects. Efficient intracellular drug release was confirmed by a pronounced biological effect on the target pathway, indicating the successful delivery of bioactive TG554 to its intracellular site of action.

The batch records developed here provide a solid foundation for future AcDex formulations and may be readily adapted to other hydrophobic drugs. Taken together, these findings highlight the potential of both TG554 and the AcDex nanoparticle platform as highly promising materials for next-generation anti-inflammatory nanomedicines. Further preclinical *in vivo* studies will be essential to investigate pharmacokinetic and therapeutic efficacy of this system under physiological conditions to advance this formulation toward potential clinical translation.

## Conflicts of interest

The authors declare no competing interests.

## Supplementary Material

RA-016-D6RA01745B-s001

## Data Availability

The data supporting this article are available in the supplementary information (SI). Supplementary information: including polymer synthesis and characterization data, nanoparticle formulation records, physicochemical characterization (DLS, ELS, SEM), chromatographic data for drug quantification, stability and degradation studies, *in vitro* assays, flow cytometry, and UPLC–MS/MS-based metabololipidomics. See DOI: https://doi.org/10.1039/d6ra01745b.
