# Supplementary material for: Encapsulation of the novel mPGES-1 inhibitor TG554 in acetalated dextran nanoparticles
Source: RSC Adv. 2026 May 19;16(29):26869–79. doi: 10.1039/d6ra01745b (PMC13187903; doi:10.1039/d6ra01745b)
Supplement: RA-016-D6RA01745B-s001 [file RA-016-D6RA01745B-s001.pdf]

Supplementary Information (SI) for RSC Advances.
This journal is © The Royal Society of Chemistry 2026

Supporting information

Encapsulation of the novel mPGES-1 inhibitor TG554 in acetalated dextran nanoparticles

Franziska Adermann[1,2], Jana Ismail[1,2], Thorben Köhler[1,2], Lea C. Klepsch[1,2], Bärbel Beringer-Siemers[1,2], Carolin Kellner[1,2], Steffi Stumpf[1,2], David Pretzel[1,2], Lisa Jäpel[1,2], Ivo Nischang[1,2,3,4], Zehra Tuğçe Gür Maz[5], Philipp Dahlke[6], Hannes Engelbrecht[6], Paul M. Jordan[2,6], Antje Vollrath[1,2], Stephanie Schubert[1,2], Erden Banoglu[5], Oliver Werz[2,6], Ulrich S. Schubert[1,2,3,4]*

[1]Laboratory of Organic and Macromolecular Chemistry (IOMC), Friedrich Schiller University Jena, Humboldtstraße 10, 07743 Jena, Germany

[2]Jena Center for Soft Matter (JCSM), Friedrich Schiller University Jena, Philosophenweg 7, 07743 Jena, Germany

[3]Helmholtz-Zentrum Berlin für Materialien und Energie GmbH (HZB), Hahn-Meitner-Platz 1, 14109 Berlin, Germany

[4]Helmholtz Institute for Polymers in Energy Applications Jena (HIPOLE Jena), Lessingstraße 12-14, 07743 Jena, Germany

[5]Department of Pharmaceutical Chemistry, Faculty of Pharmacy, Gazi University, 06560 Ankara, Turkey

[6]Department of Pharmaceutical/Medicinal Chemistry, Institute of Pharmacy, Friedrich Schiller University Jena, Philosophenweg 14, 07743 Jena, Germany

* Corresponding author: ulrich.schubert@uni-jena.de

1. Table of content

1. Table of content 2

2. Synthesis and characterization of methoxy acetal derivatized dextran (AcDex) 3

2.1 Materials 3

2.2 Instrumentation 3

2.3 Synthesis procedure of AcDex 4

3. Formulation of the nanoparticles 7

3.1 Materials 7

3.2 Instrumentation 8

3.3 Batch records 8

4. Characterization of the nanoparticles 12

4.1 Particle characteristics 12

4.2 HPLC analysis of drug loaded nanoparticles 15

4.3 Storage stability of nanoparticles 17

4.4 Degradation of nanoparticles 19

4.5 Biosafety and cellular uptake 22

4.6 Bioactivity 22

2. Synthesis and characterization of methoxy acetal derivatized dextran (AcDex)

2.1 Materials

Dextran from *Leuconostoc mesenteroides* (average molar mass 9.000 to 11.000 g mol^{-1}, Sigma-Aldrich) was lyophilized (-55 °C, 3 mbar, 2 days) prior to usage. Pyridinium *p*-toluenesulfonate (PPTS, 98 %, Sigma-Aldrich), 2-methoxy-propene (97 %, Sigma-Aldrich), dimethyl sulfoxide (DMSO, 99.7 %, extra dry, over molecular sieves, AcroSeal®, Acros Organics) triethylamine (Et_3N, 99 %, Thermo Scientific), dimethylsulfoxid-d6 (DMSO-d6, 99,8 % D, Eurisotop), deuterium oxide (D_2O, 99.90 % D, Eurisotop) and deuterium chloride (DCl, solution 35 % (w/w) in D_2O, 99 % D, Sigma-Aldrich) were all used without further purification. Deionized water (diH_2O) was purified from tap water using a DI2000 mixed-bed water demineralizer from Thermo Scientific. Acetone (technical quality) was purified by distillation prior to usage.

2.2 Instrumentation

Proton nuclear magnetic resonance (^{1}H-NMR) spectra were recorded using a Bruker Avance NEO 300 (300.13 MHz, ^{1}H; 75.5 MHz, ^{13}C) with a 5 mm PA BBO 300S1 BBF-H-D-05Z probe head and a SampleJet robot for automated high-throughput sample processing. The samples were measured without shimming (64 scans at 297 K) in Bruker NMR 7" tubes with coded closed caps.

For the intact polymer, the sample was prepared by dissolving 11 mg of the polymer in 570 µL of DMSO-d6, then the solution was transferred to a NMR tube and measured. For degree of substitution (DS) calculation, the sample was prepared by suspending 14 mg of the polymer in 570 µL D_2O in a 5 mL glass vial with a lid. Three drops of DCl were added, and the sample was shaken for 6 min. The solution of the degradation products was transferred into a NMR-tube, and the measurement was initiated exactly 10 min after the addition of DCl.

The DS is defined as the number of hydroxyl groups replaced by a given functionalization per AGU and can have a maximum value of three in total. To determine the DS of AcDex, the integral of the anhydroglucose unit (AGU) (δ = 3.05 to 3.70 ppm; without the anomeric center) was normalized to 6.0. The integral of methanol (δ = 2.95 ppm) divided by three gives the acyclic DS. The integral of acetone (δ = 1.83 ppm) divided by six, subtracted by the acyclic DS and multiplied by two (number of hydroxy groups replaced per cyclic substitution) results in the cyclic DS.

$$DS_{cycl} = 2\left(\frac{\int Acetone}{6} - DS_{acycl}\right) \text{ with } DS_{acycl} = \frac{\int MeOH}{3}$$

Size exclusion chromatography (SEC) elugrams were recorded using an Agilent 1200 series system (degasser: PSS, pump: G1310A, autosampler: G1329A, Diode Array Detector (DAD): G1315D, Refractive Index Detector (RID): G7162A) from Agilent Technologies running an isocratic solvent of 0.21 % (w/w) LiCl in DMAc. The measurements were performed after an injection of 50 µL over a period of 30 min with a flowrate of 1 mL min^{-1}. The columns (PSS GRAM guard/30/1,000 Å, 10 µm particle size) were stored in a column oven (Techlab) and tempered to 40 °C. The molar mass and dispersity were calculated based on RI-detection and a polystyrene calibration (Polymer Standard poly(styrene), 375 to 1,040,000 g mol^{-1}, PSS).

The sample was prepared by dissolving approx. 4 mg of the polymer in a solution of 0.21 % (w/w) LiCl in DMAc to a concentration of 2 mg mL^{-1}. The sample was filtered through a 0.45 µm PTFE-filter (13 mm, AppliChrom) prior to measurement.

2.3 Synthesis procedure of AcDex

Lyophilized dextran (10 kDa to 1.50 g, 9.26 mmol AGU) and 0.012 equiv. PPTS (0.028 mg, 0.111 mmol) were dissolved in 15 mL dry DMSO (70 ppm H_2O, determined via Karl Fischer titration) under argon-atmosphere. 6 equiv. (2 equiv. per hydroxyl group) 2-methoxypropene (5.32 mL, 55.6 mmol) were added. After 60 min, the reaction mixture was split into two equal volumes, each quenched with 1 mL triethylamine (TEA, Et_3N, shaken for 2 min), precipitated by addition of 40 mL diH_2O, centrifuged (10.000 rpm, 10 °C, 20 min), decanted and lyophilized for two days. For purification, the dry white powder batches were combined and dissolved in 3 mL acetone, precipitated by addition of 45 mL basic-diH_2O (0.02 v % TEA in diH_2O), centrifuged (10.000 rpm, 10 °C, 10 min), decanted and lyophilized for three days.

The reaction yielded 2.048 g of purified AcDex (DS_{acycl} = 0.46; DS_{cycl} = 1.97).

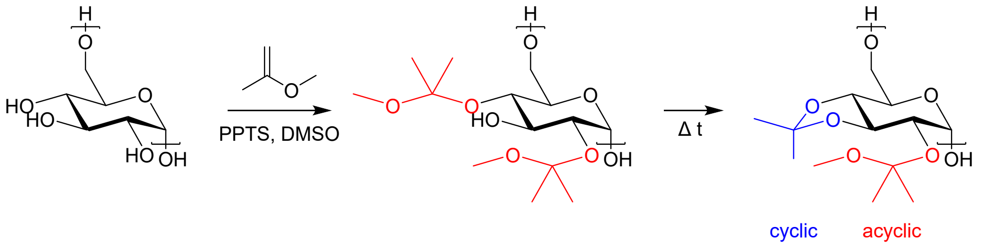

Figure S1: Schematic representation of the acetalization of dextran yielding AcDex.

Centrifugation was performed using a Hettich Rotina 380R centrifuge in 50 mL centrifuge vials (Cellstar® tubes 50 mL, Greiner Bio-one). Lyophilization was performed using a CHRIST Alpha 1-2 LD freeze-dryer at −78 °C and 0.082 mbar, unless otherwise stated.

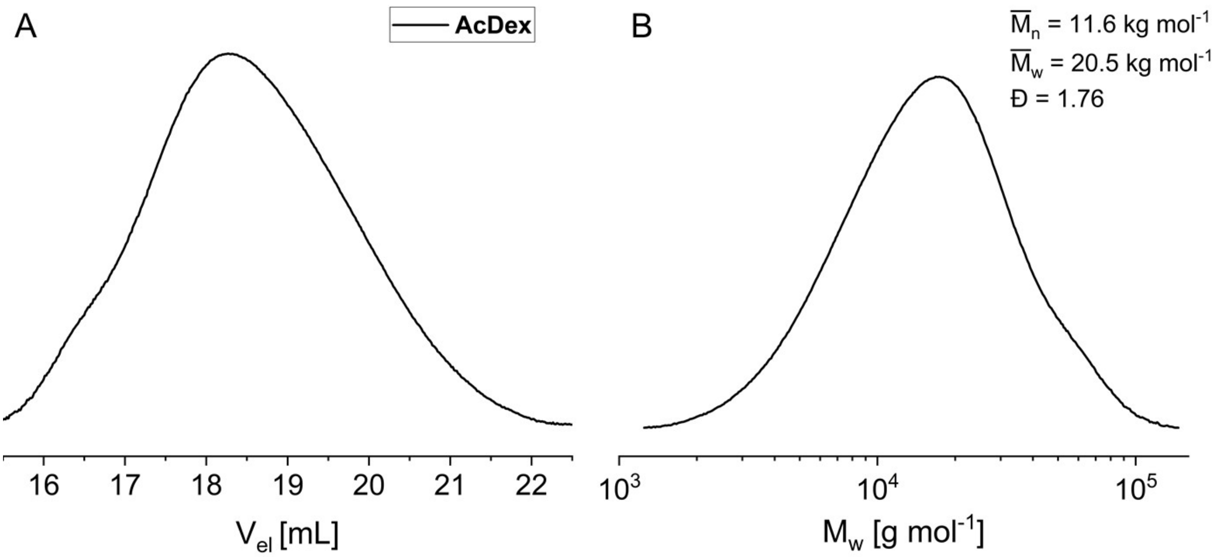

Figure S2: SEC of AcDex (Eluent 0.21 % (w/w) LiCl in DMAc, RID, PS calibration). (A) Elugram, (B) molar mass distribution.

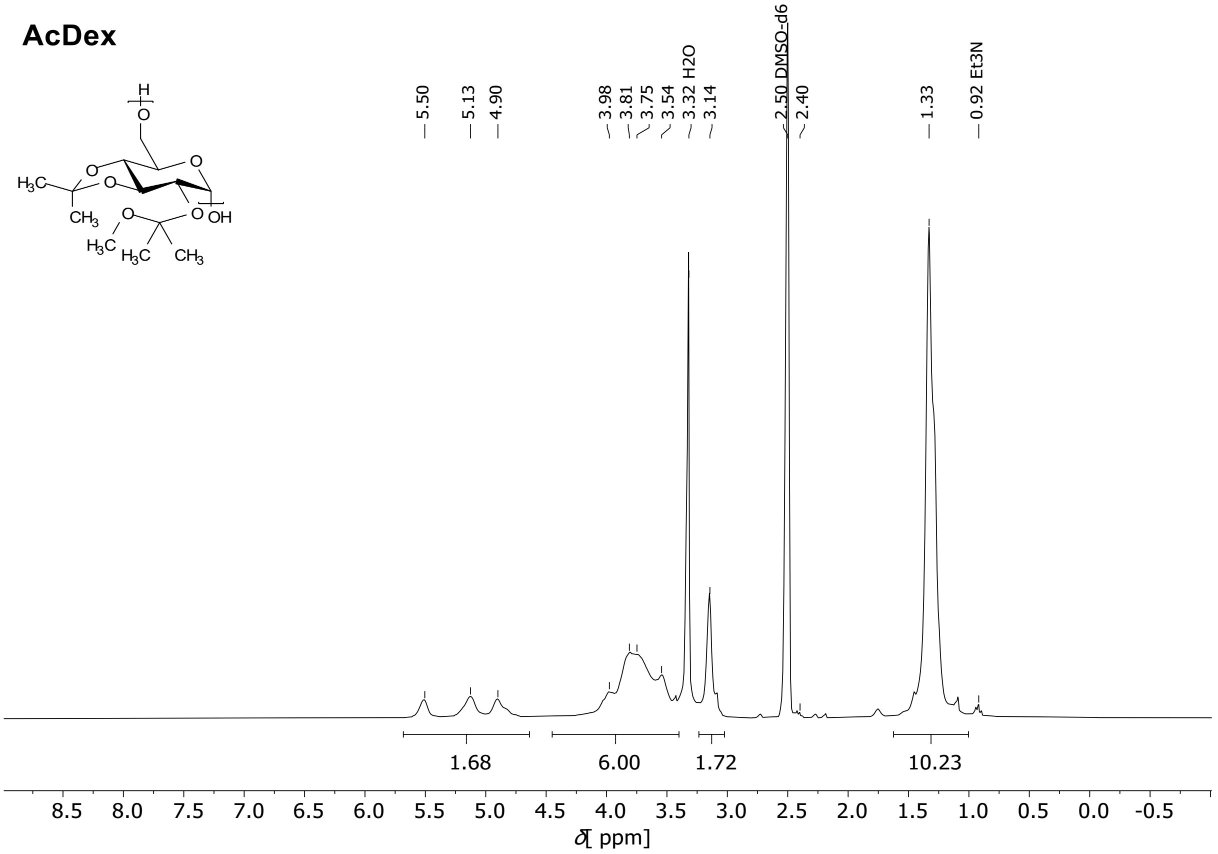

Figure S3: ^{1}H-NMR spectrum (DMSO-d6, 300 MHz) of AcDex.

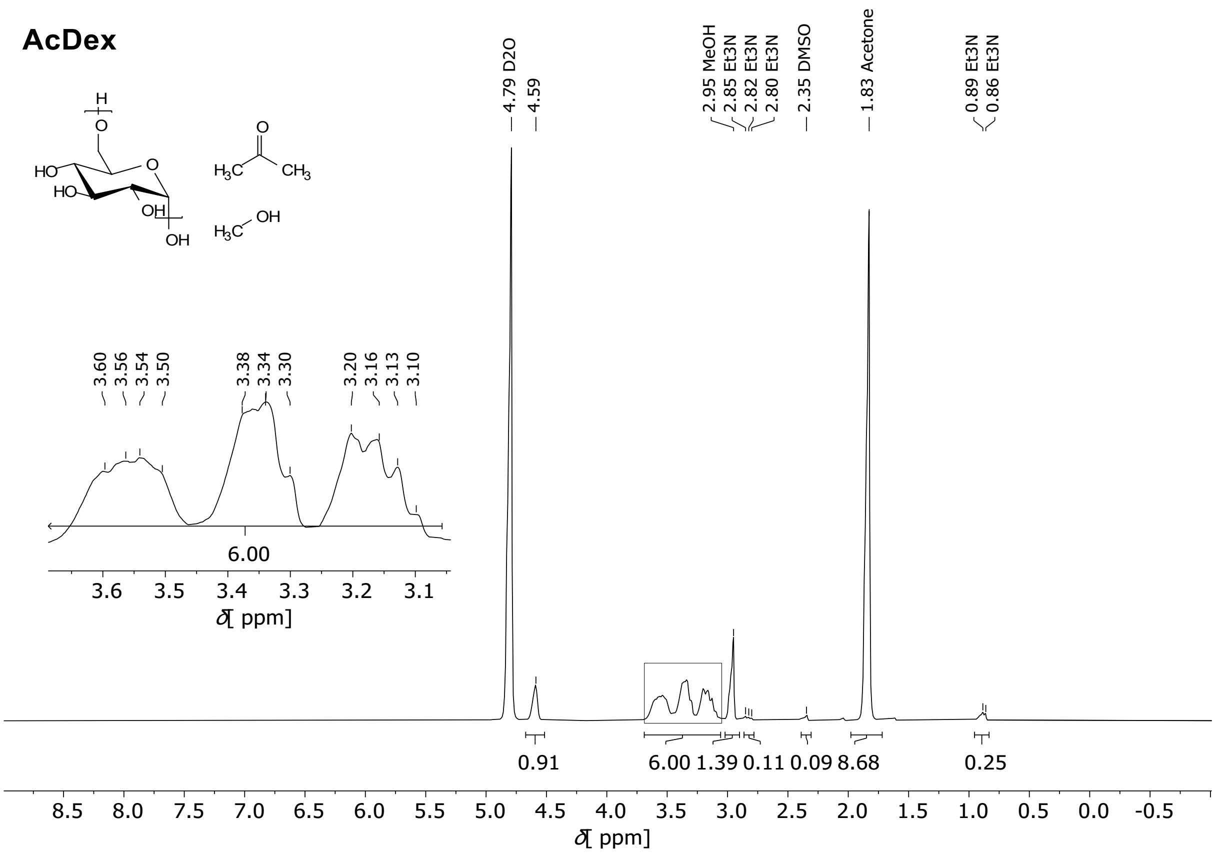

Figure S4: ^{1}H-NMR spectrum (D_2O with DCl, 300 MHz) of AcDex (degradation products) measured 10 min after the addition of DCl.

3. Formulation of the nanoparticles

3.1 Materials

Table S1: Supplier and information for the materials used for the production of AcDex[Blank] and AcDex[TG554] nanoparticles.

Material	Supplier and details
Acetone	Acros, Thermo Scientific
DMSO, anhydrous ≥99.9 %)	Sigma Aldrich
Poly(vinyl alcohol) (PVA) Parteck® MXP PVA 4-88	Sigma Aldrich
Triethylamine (TEA, Et_3N, 99 %)	Thermo Scientific
Sterile water	Merck
AcDex	Synthesized (see SI 2.0)
TG554	Synthesized see Ref [5]

Table S2: Materials for production of AcDex[Blank] nanoparticles.

Material	Required amount per batch
AcDex (polymer)	20 mg
Acetone (solvent for polymer)	2 mL
DMSO (solvent for drug)	0.375 mL
Sterile water (medium)	20 mL
TEA 99 % (additive)	50 µL
Poly(vinyl alcohol) (PVA, surfactant)	0.3 g

Table S3: Materials for production of AcDex[TG554] nanoparticles.

Material	Required amount per batch
AcDex (polymer)	20 mg
TG554 (drug)	1.5 mg
Acetone (solvent for polymer)	2 mL
DMSO (solvent for drug)	0.375 mL
Sterile water(medium)	20 mL
TEA 99 % (additive)	50 µL
PVA (surfactant)	0.3 g

3.2 Instrumentation

Table S4: Instruments and equipment used for the production of AcDex[TG554] nanoparticles.

Equipment	Supplier / Specifications
Fine balance	Mettler Toledo XPR
Syring pump	Aladdin AL-1000, flow range 0.001 µL h^{-1} to 3470 mL h^{-1}
Stirring plate	2mag MIX 15 eco multi-position magnetic stirrer
Magnetic stirrer with heating plate	Heidolph heating and magnetic stirrers MR Hei-Standard
Centrifuge	Eppendorf centrifuge 5804 R<br>Rotor FA-45-6-30
Ultrasonic bath	BANDELIN SONOREX SUPER RK 514 H ultrasonic bath with heater
Vortexer	IKA Vortex 2
Pipettes	Eppendorf Research® plus 10 mL, 5 mL, 1 mL, 0.2 mL, 10 µL
Large glass beaker	VWR®, standard glass beakers
Glass vials, snap cap	VWR®, Snap Cap Vials 5 mL,10 mL, 20 mL
Stirring bars	VWR®, Size 2 to 5 mm wide, 13 to 15 mm long, sterilized with ethanol
Spatula	Standard metal spatula, sterilized with ethanol
Float for ultrasonic devices	Heathrow Scientific floating foam microtube rack, tube size 1.5/2.0 mL<br>Heathrow Scientific floating foam microtube rack, tube size 50 mL
Sterile syringe filters	Carl Roth® syringe filters hydrophobic polytetrafluoroethylene (PTFE), 0.22 µm, 13 mm, sterile<br>TH. GYER LABSOLUTE® syringe filters cellulose acetate (CA), 0.22 µm, sterile
Single use syringes	B.Braun Injekt® Luer Solo 2 mL
Cannulas	B.Braun Sterican® cannula, green, 21G (gauge = 0.80 mm) outer diameter, length 120 mm, sterile
Lyo vials	VWR®, 2 mL shell vial 12x31 mm, clear glass, 12 mm PE-Plug
Falcon tubes	Corning®, 50 mL centrifuge tubes, sterile
Eppendorf tubes 5 mL	Eppendorf Tubes® 5 mL, sterile
Microtubes	Axygen® 1.5 mL MaxyClear Microtubes MCT-150-C

3.3 Batch records

Table S5: Batch record for the production of AcDex[Blank] nanoparticles.

Pos	Amount	Material / Process	Details / Description of the workflow
**Preparation of the polymer solution**			
1	20 mg	AcDex	Weigh in the substance in a 5 mL Eppendorf tube on a fine balance.
2	2.0 mL	Aceton	Measure the required volume of the solvent acetone and add it to the AcDex to dissolve the polymer. Filter the polymer solution through a sterile PTFE filter.
3	8.1 mL	Sterile water	Measure the required volume of sterile water and fill it into a 20 mL glass vial.
4			Place the glass vial on a stirring plate and add a stirring bar to the vial.
5	2.0 mL	PVA 3 %	Sterile filter PVA solution 3 % through a sterile filter.

Pos	Amount	Material / Process	Details / Description of the workflow
6	0.9 mL	PVA 3 %	Measure the required volume of the PVA solution and add the amount to the glass vial with the sterile water (final PVA concentration 0.3 % (w/w)).
7	1.5 mL	TEA 0.1 %	Sterile filter TEA 0.1 % solution through a PTFE filter.
8	0.9 mL	TEA 0.1 %	Measure the required volume of the TEA solution and add the amount to the sterile water containing PVA.
**Formulation**			
9			Place the syringe pump close to the vial on a stirring plate.
10			Attach the cannula to the syringe, remove the protective cap.
11	1.5 mL	AcDex solution	Draw the polymer solution into a syringe with a cannula, remove any air bubbles if necessary.
12			Insert the syringe into the syringe pump. Bend the cannula so that the tip of the needle is immersed in the sterile water/PVA/TEA medium.
13			Switch on the stirring plate and stir at 800 rpm.
14			Adjust the needle of the syringe so that it is halfway up to the vortex. Start at the edge of the vortex so that the solution is not in the middle of vortex, but just before it, so that the injected solution is distributed immediately.
15			Program the syringe pump to a syringe diameter of 10 mm and a speed of 2 mL min^{-1}.
16		Formulation	Start the program and the injection of the polymer solution (organic phase) into the sterile water/PVA/TEA (aqueous phase).
17			When the syringe is empty, carefully remove it and dispose of the syringe and the needle.
18	20 h	Solvent evaporation	Allow the dispersion to stir for 20 h (overnight) to evaporate the solvent.
19	-	-	Turn off the stirring plate, leave the vial on it.
**First quality control via DLS**			
20	0.1 mL particle dispersion	First DLS quality control	Fill 0.1 mL of the prepared nanoparticle dispersion in a 0.5 mL tube and measure DLS according to SOP.
21	~10 mL	Dispersion	Transfer the rest of the dispersion to a 50 mL falcon tube and close tightly.
**Purification of the nanoparticles**			
22	3.0 mL	Sterile water	Prepare a fresh TEA solution with 0.01 % TEA by measuring the required volume sterile water and adding it in a 10 mL glass vial.
23	0.3 mL	TEA	Measure the required volume of the TEA solution and add it to sterile water.
24		Centrifugation	Centrifuge the nanoparticle dispersion at 11,000 rpm for 60 min at 20 °C. A nanoparticle pellet should be visible after the centrifugation on the bottom.
25			Remove the supernatant in the falcon very carefully. Do not resuspend the particle pellet on the bottom of the falcon tube while removing the supernatant.
**Resuspension & storage of the nanoparticles**			

Pos	Amount	Material / Process	Details / Description of the workflow
26	3.0 mL	0.01 % TEA Solution	Add the prepared sterile 0.01 % TEA water to the nanoparticle sediment in the falcon tube and resuspend the particles by pipetting up and down at least 10 times with a 1 mL pipette.
27		Resuspension	Vortex the falcon tube for 30 s
28			Put the falcon tube with the sample in an ultrasonication bath for 15 min at 20 °C.
29		Storage	Store the resuspended sample in the falcon tube overnight at 4 °C in the refrigerator for further equilibration.
30		Equilibrate	Remove the sample in the falcon tube from the refrigerator and allow it to equilibrate to room temperature for 10 min.
31		Mixing	Swivel the falcon and vortex it for 10 s to ensure thorough mixing.
32		DLS	Use 50 µL of the sample in the required cuvette and measure DLS as quality control after purification according to SOP.
33		Filling	Fill the product into a sterile 5 mL tube or leave it in the falcon tube. Store the sample in the fridge at 4 °C until further analysis.

Table S6: Batch record for the production of AcDex[TG554] nanoparticles.

Pos	Amount	Material / Process	Details / Description of the workflow
**Preparation of the drug solution**			
1		TG554	Remove the stored drug from the refrigerator (4 °C) and allow it to equilibrate to room temperature for 10 min protected against light.
2	1.5 mg	Drug TG554	Weigh the substance on the fine balance in a microtube.
3	0.375 mL	DMSO	Measure the required volume of the solvent DMSO and add it to the microtube that contains the drug TG554. The final drug concentration of the stock solution must be 4 mg mL^{-1}.
4		TG554 in DMSO	Vortex the tube containingTG554 in DMSO for 10 s.
5			Apply 15 min ultrasonication at 25 °C to the sample.
6			Vortex the tube again for 10 s.
7			Filter the TG554 solution through a sterile PTFE filter.
**Preparation of the polymer solution**			
8	20 mg	AcDex	Weigh the substance in a 5 mL Eppendorf tube on the fine balance.
9	2.0 mL	Aceton	Measure the required volume of the solvent acetone and add it to the AcDex to dissolve the polymer. Filter the polymer solution through a sterile PTFE filter.
10	8.1 mL	Sterile water	Measure the required volume of sterile water and fill it into a 20 mL glass vial.
11			Place the glass vial with the water on a stirring plate and add a stirring bar to the vial.
12	2.0 mL	PVA 3 %	Filter the PVA solution 3 % through a sterile filter.
13	0.9 mL	PVA 3 %	Measure the required volume of the PVA solution and add the amount to the glass vial with the sterile water (final PVA

Pos	Amount	Material / Process	Details / Description of the workflow
			concentration 0.3 % (w/w)).
14	1.5 mL	TEA 0.1 %	Filter the TEA 0.1 % solution through a sterile filter.
15	0.9 mL	TEA 0.1 %	Measure the required volume of the TEA solution and add the amount to the sterile water containing PVA.
**Formulation**			
16	112.5 µL	TG554 in DMSO	Add the required amount of TG554 solution to the AcDex polymer solution in the 5 mL glass vial (corresponds to 3 % (w/w) drug load based on AcDex).
17			Close the glass vial and vortex it for 10 s.
18			Place the syringe pump close to the vial on a stirring plate.
19			Attach the cannula to the syringe, remove the protective cap.
20	1.613 mL	AcDex & TG554 solution	Draw the polymer solution into a syringe with a cannula, remove any air bubbles if necessary.
21			Insert the syringe into the syringe pump. Bend the cannula so that the tip of the needle is immersed in the sterile water/PVA/TEA medium.
22			Switch on the stirring plate and stir at 800 rpm.
23			Adjust the needle of the syringe so that it is halfway up to the vortex. Start at the edge of the vortex so that the solution is not in the middle of vortex, but just before it, so that the injected solution is distributed immediately.
24			Program the syringe pump to a syringe diameter of 10 mm and a speed of 2 mL min^{-1}.
25		Formulation	Start the program and the injection of the polymer solution (organic phase) into the sterile water/PVA/TEA (aqueous phase).
26			When the syringe is empty, carefully remove it and dispose of the syringe and the needle.
27	20 h	Solvent evaporation	Allow the dispersion to stir for 20 h (overnight) to evaporate the solvent.
28	-	-	Turn off the stirring plate, leave the vial on it.
**First quality control via DLS**			
29	0.1 mL particle dispersion	First DLS quality control	Fill 0.1 mL of the prepared nanoparticle dispersion in a 0.5 mL tube and measure DLS according to SOP.
30	~10 mL	Dispersion	Transfer the rest of the dispersion to a 50 mL falcon tube and close tightly.
**Purification of the TG554 loaded nanoparticles**			
31	3.0 mL	Sterile water	Prepare a fresh TEA solution with 0.01 % TEA by measuring the required volume sterile water and adding it in a 10 mL glass vial.
32	0.3 mL	TEA	Measure the required volume of the TEA solution and add it to sterile water.
33		Centrifugation	Centrifuge the nanoparticle dispersion at 11,000 rpm for 60 min at 20 °C. A nanoparticle pellet should be visible after the centrifugation on the bottom.
34			Remove the supernatant in the falcon very carefully. Do not resuspend the particle pellet on the bottom of the falcon tube while

Pos	Amount	Material / Process	Details / Description of the workflow
			removing the supernatant.
**Resuspension & storage of the TG554 loaded nanoparticles**			
35	3.0 mL	0.01 % TEA Solution	Add the prepared sterile 0.01 % TEA water to the nanoparticle sediment in the falcon tube and resuspend the particles by pipetting up and down at least 10 times with a 1 mL pipette.
36		Resuspension	Vortex the falcon tube for 30 s
37			Put the falcon tube with the sample in an ultrasonication bath for 15 min at 20 °C.
38		Storage	Store the resuspended sample in the falcon tube overnight at 4 °C in the refrigerator for further equilibration.
39		Equilibrate	Remove the sample in the falcon tube from the refrigerator and allow it to equilibrate to room temperature for 10 min.
40		Mixing	Swivel the falcon and vortex it for 10 s to ensure thorough mixing.
41		DLS	Use 50 µL of the sample in the required cuvette and measure DLS as quality control after purification according to SOP.
42		Filling	Fill the product into a sterile 5 mL tube or leave it in the falcon tube. Store the sample in the fridge at 4 °C until further analysis.

4. Characterization of the nanoparticles

4.1 Particle characteristics

Table S7: Determined characteristics of the individual formulations.

Sample	d_h [a] [nm]	PDI [a]	ζ [b] [mV]	$c_{Nanoparticle}$ [c] [mg mL^{-1}]	LC [d] [%]	EE [d] [%]	Yield [e] [%]
**AcDex[Blank] n = 1**	158	0.074	−22	3.61	—	—	72
**AcDex[Blank] n = 2**	186	0.140	−23	3.31	—	—	66
**AcDex[Blank] n = 3**	161	0.131	−19	3.57	—	—	71
**AcDex[TG554] n = 1**	165	0.090	−16	3.35	2.28	76	65
**AcDex[TG554] n = 2**	163	0.083	−18	3.39	2.11	70	66
**AcDex[TG554] n = 3**	160	0.041	−19	3.73	2.18	73	72

a) Hydrodynamic diameter (d_h, z-average) and polydispersity index (PDI) of purified particles determined by DLS.
b) Zeta potential (ζ) in ultrapure water determined by ELS.
c) Particle concentration after purification determined by weighing the freeze-dried particles.
d) Loading capacity (LC) and encapsulation efficiency (EE) determined by HPLC.
e) Yield after purification determined by dividing the actual obtained particle mass by the theoretically possible particle mass multiplied by 100.

Table S8: Determined mean characteristics of formulations ± standard deviation (SD). n = 3 for different batches of manufactured nanoparticles.

Sample	d_h [a] [nm]	PDI [a]	ζ [b] [mV]	$c_{Nanoparticle}$ [c] [mg mL^{-1}]	LC [d] [%]	EE [d] [%]	Yield [e] [%]
**AcDex[Blank] mean**	168 ± 15	0.115 ± 0.036	−21 ± 2	3.5 ± 0.16	—	—	70 ± 3
**AcDex[TG554] mean**	163 ± 3	0.071 ± 0.027	−18 ± 2	3.49 ± 0.21	2.19 ± 0.09	73 ± 3	68 ± 4

a) Hydrodynamic diameter (d_h, z-average) and polydispersity index (PDI) of purified particles determined by DLS.
b) Zeta potential (ζ) in ultrapure water determined by ELS.
c) Particle concentration after purification determined by weighing the freeze-dried particles.
d) Loading capacity (LC) and encapsulation efficiency (EE) determined by HPLC.
e) Yield after purification determined by dividing the actual obtained particle mass by the theoretically possible particle mass multiplied by 100.

Table S9: Determined characteristics of formulations with the fluorescent dye NLO for cytocompatibility and uptake studies.

Sample	d_h [a] [nm]	PDI [a]	ζ [b] [mV]	$c_{Nanoparticle}$ [c] [mg mL^{-1}]	LC [d] [%]	EE [d] [%]	Yield [e] [%]
**AcDex[NLO] n = 1**	146	0.072	−17	3.96	—	—	79
**AcDex[NLO] n = 2**	152	0.034	−14	4.11	—	—	82
**AcDex[NLO] n = 3**	180	0.120	−19	3.92	—	—	78
**AcDex[TG554/ NLO] n = 1**	172	0.053	−22	4.31	2.80	93	84
**AcDex[TG554/ NLO] n = 2**	170	0.044	−25	4.18	2.90	96	81
**AcDex[TG554/ NLO] n = 3**	163	0.059	−19	3.75	2.68	89	73

a) Hydrodynamic diameter (d_h, z-average) and polydispersity index (PDI) of purified particles determined by dynamic light scattering.
b) Zeta potential (ζ) in ultrapure water determined by electrophoretic light scattering.
c) Particle concentration after purification determined by weighing the freeze-dried particles.
d) Loading capacity (LC) and encapsulation efficiency (EE) determined by HPLC.
e) Yield after purification determined by dividing the actual obtained particle mass by the theoretically possible particle mass multiplied by 100.

Table S10: Determined mean characteristics of formulations ± SD for cytocompatibility and uptake studies. n = 3 for different batches of manufactured nanoparticles.

Sample	d_h [a] [nm]	PDI [a]	ζ [b] [mV]	$c_{Nanoparticle}$ [c] [mg mL^{-1}]	LC [d] [%]	EE [d] [%]	Yield [e] [%]
**AcDex[NLO] mean**	159 ± 18	0.075 ± 0.043	−17 ± 2	4.00 ± 0.10	—	—	80 ± 2
**AcDex[TG554/ NLO] mean**	168 ± 4	0.052 ± 0.008	−22 ± 3	4.08 ± 0.29	2.79 ± 0.11	93 ± 3	79 ± 6

a) Hydrodynamic diameter (d_h, z-average) and polydispersity index (PDI) of purified particles determined by dynamic light scattering.
b) Zeta potential (ζ) in ultrapure water determined by electrophoretic light scattering.
c) Particle concentration after purification determined by weighing the freeze-dried particles.
d) Loading capacity (LC) and encapsulation efficiency (EE) determined by HPLC.
e) Yield after purification determined by dividing the actual obtained particle mass by the theoretically possible particle mass multiplied by 100.

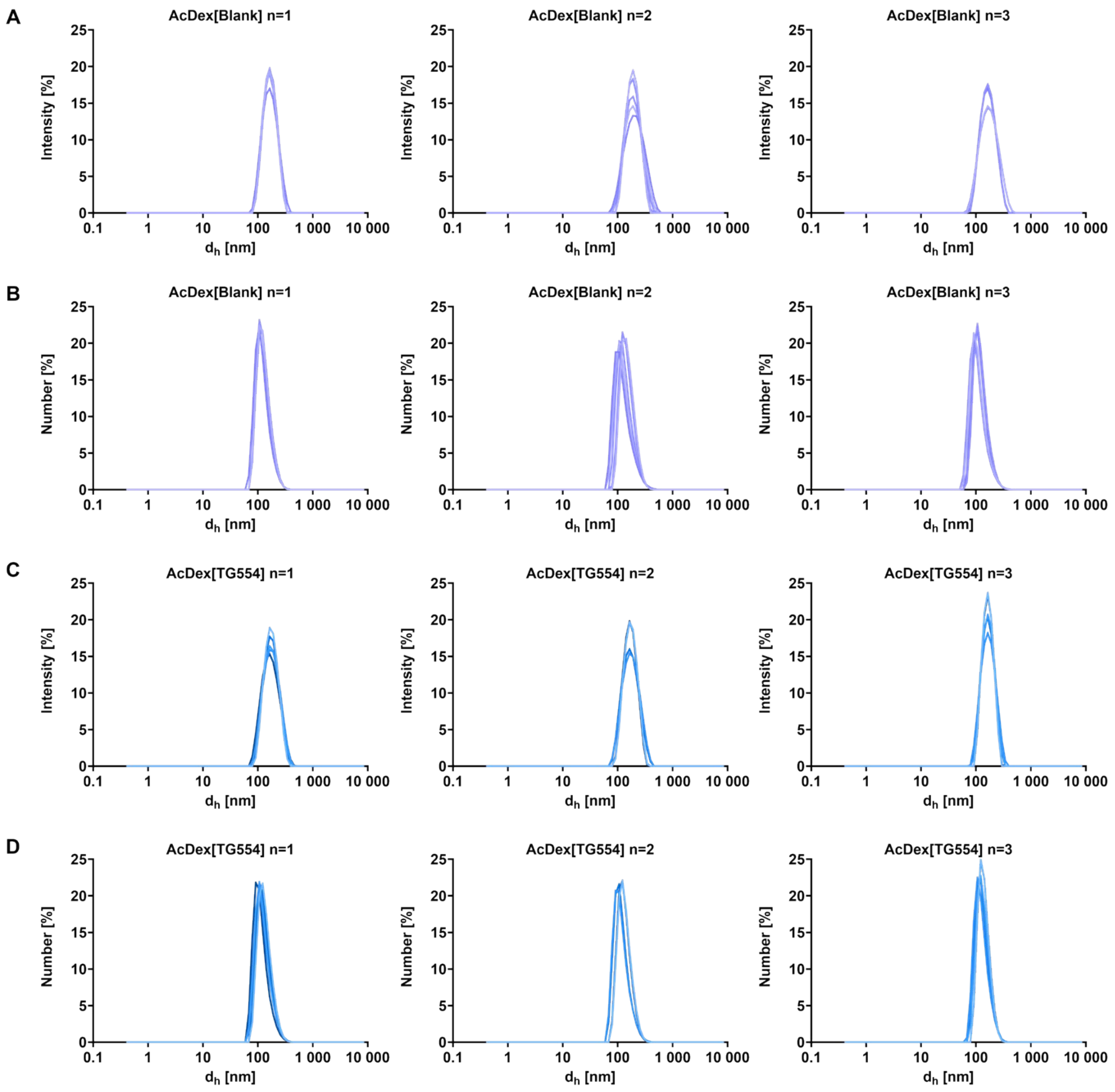

Figure S5: Nanoparticle size distributions (z-average value represented as d_h) obtained by dynamic light scattering (five measurements per individual formulation represented as overlay in each size distribution plot). n = 3 different batches of manufactured nanoparticles. (A) intensity and (B) number of AcDex[Blank] nanoparticle formulations and (C) intensity and (D) number of AcDex[TG554] nanoparticle formulations.

4.2 HPLC analysis of drug loaded nanoparticles

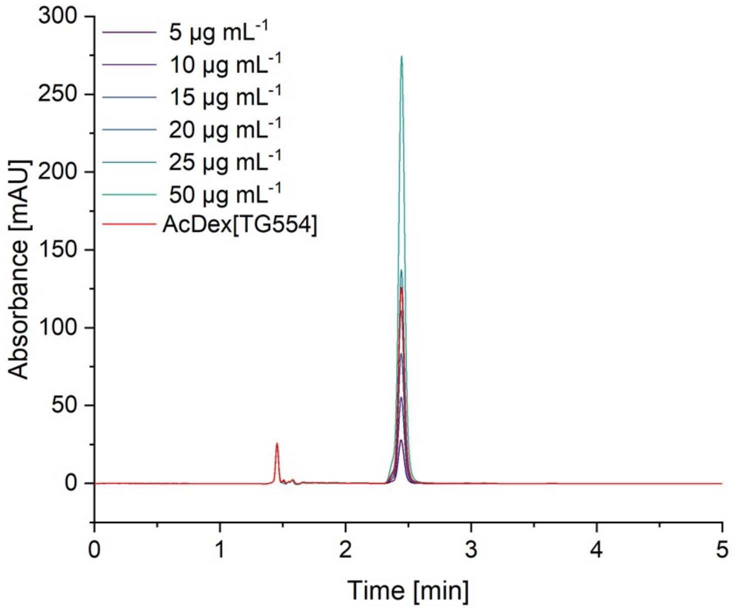

Figure S6: Elution of varying concentrations of solutions of TG554 as well as a dissolved AcDex[TG554] nanoparticle population monitored by the Diode Array Detector (DAD) at 287 nm. Apparently, the presence of polymer (not visible in UV) does not interfere with the elution time and profile of the drug. The injection volume was 5 µL and a flow rate of 1 mL min^{-1} was utilized. The binary mobile phase solvent composition consisted of CH_3CN and 0.1 % formic acid (FA) in water (v/v). Drug elution occurred during the isocratic hold at 70/30 CH_3CN/0.1% aqueous FA (%, v/v) within the first 5 min.

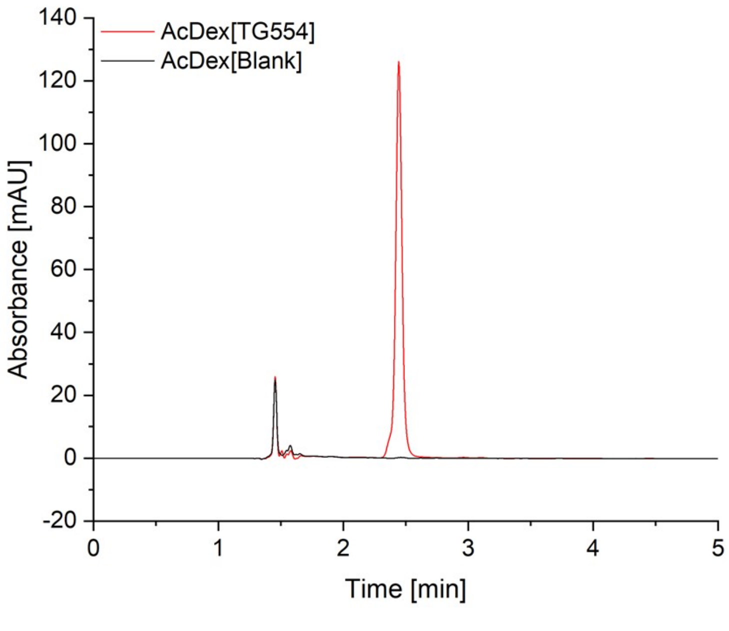

Figure S7: Elution of dissolved AcDex[TG554] and AcDex[Blank] monitored at a wavelength of 287 nm with DAD demonstrating no interference of the polymer in detection of the drug. Same chromatographic conditions as in Figure S6.

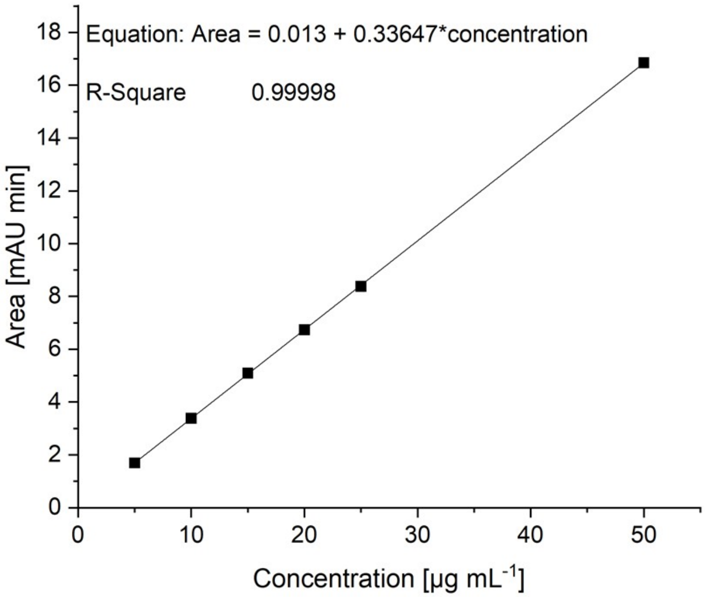

Figure S8: Calibration curve obtained from integration of peaks corresponding to varying amounts of drug obtained from Figure S6.

4.3 Storage stability of nanoparticles

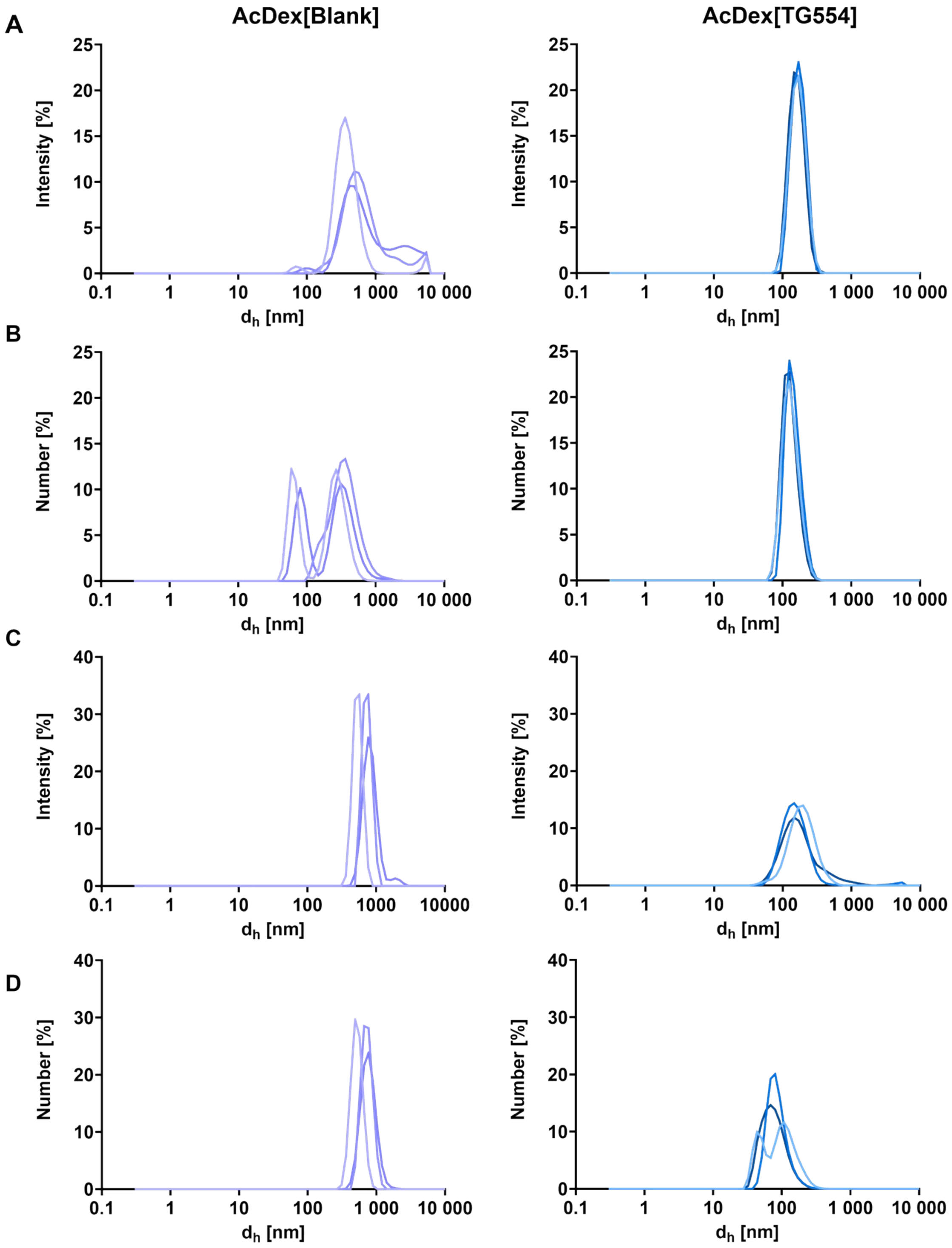

Figure S9: Mean nanoparticle size (z-average value represented as d_h) distribution by (A) intensity and (B) number distributions of AcDex[Blank] and AcDex[TG554] nanoparticles of n = 3 independently formulated particle systems after three months of storage at 4 °C obtained by dynamic light scattering. Mean nanoparticle size (z-average value represented as d_h) distribution by (C) intensity and (D) by number after 6 months of storage at 4 °C.

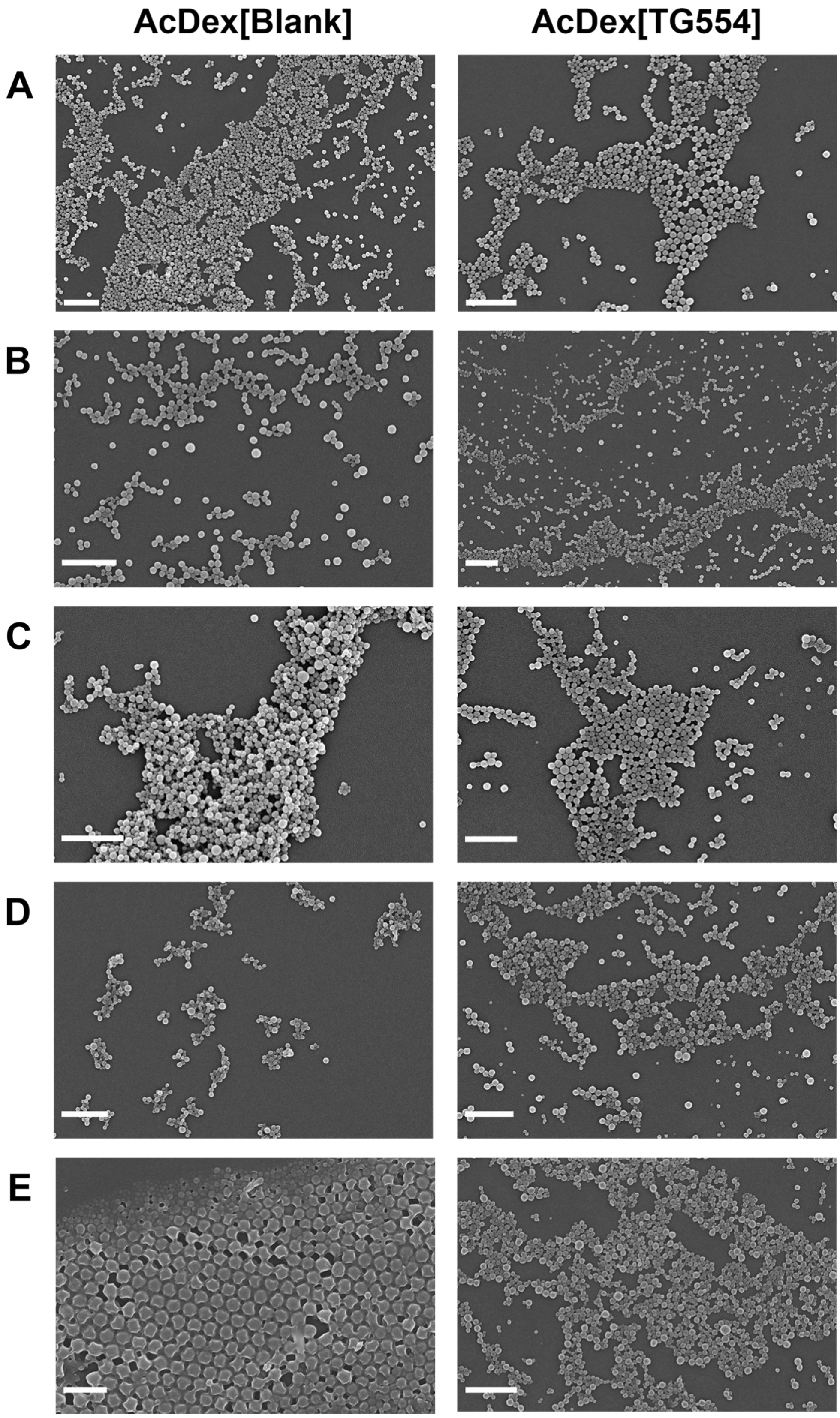

Figure S10: Scanning electron microscopy images of AcDex[Blank] and AcDex[TG554] nanoparticles after formulation (A = 0 months) and after several months of storage (B = 1 month, C = 2 months, D = 3 months, E = 4 months) at 4 °C. Scale bar = 1 µM.

4.4 Degradation of nanoparticles

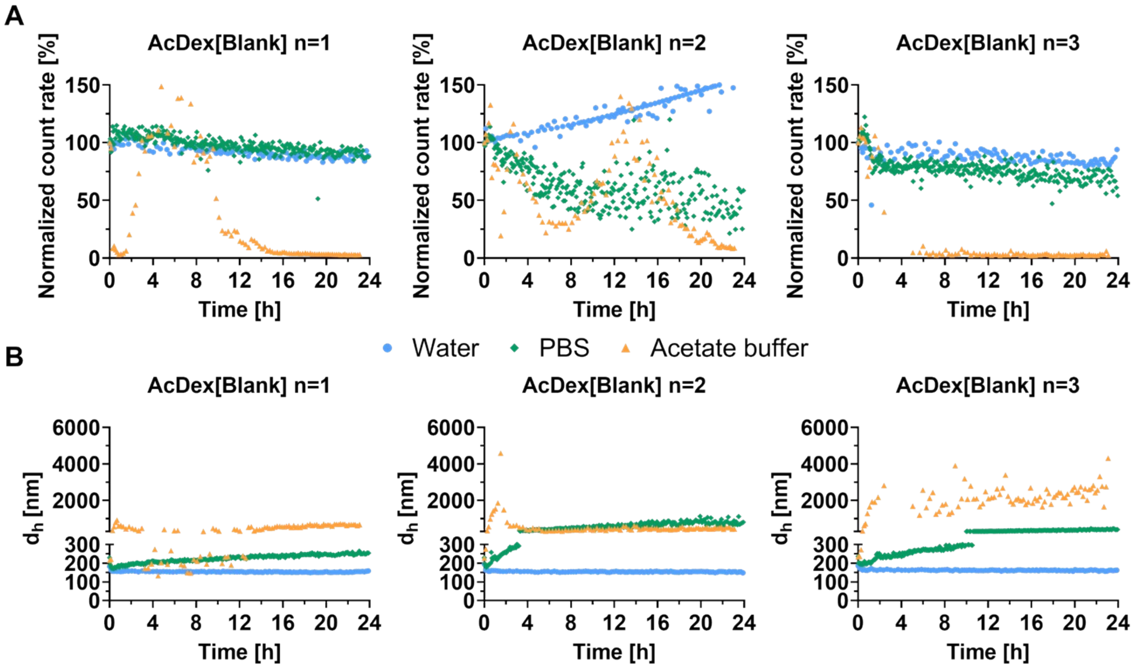

Figure S11: Degradation profile of AcDex[Blank] particles using different media. Degradation was observed by monitoring the (A) count rate and (B) size (z-average value represented as d_h) by dynamic light scattering over time in ultrapure water, PBS and acetate buffer. n = 3 different batches of manufactured nanoparticles.

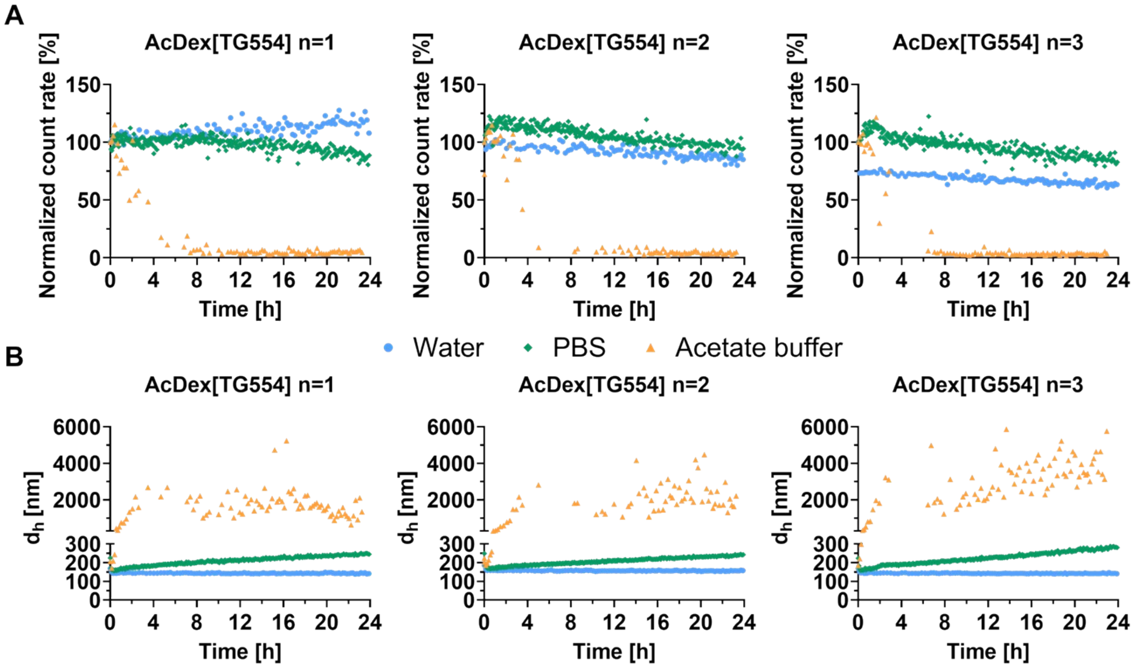

Figure S12: Degradation profile of AcDex[TG554] particles using different media. Degradation was observed by monitoring the (A) count rate and (B) size (z-average value represented as d_h) by dynamic light scattering over time in ultrapure water, PBS and acetate buffer. n = 3 different batches of manufactured nanoparticles.

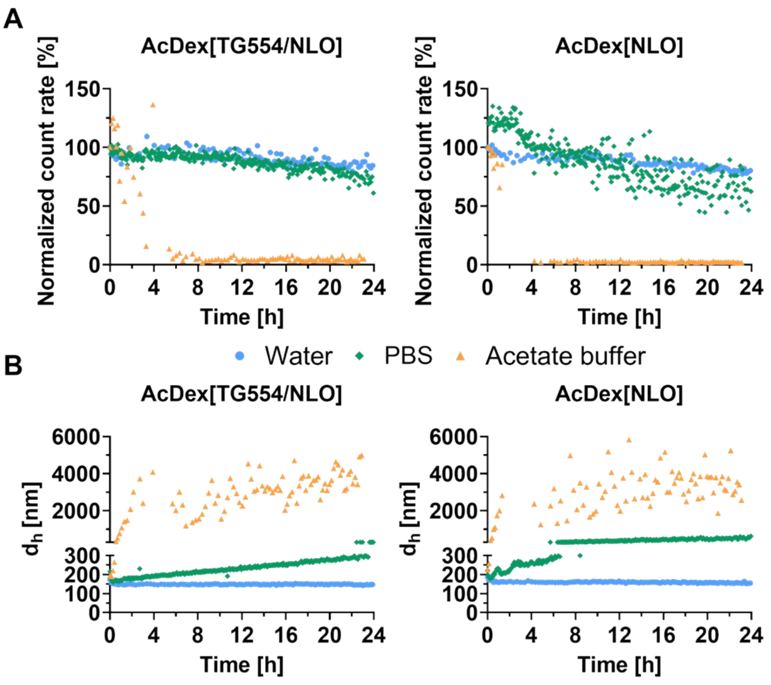

Figure S13: Degradation profile of AcDex[NLO] and AcDex[TG554/NLO] particles using different media. Degradation was observed by monitoring the (A) count rate and (B) size (z-average value represented as d_h) by dynamic light scattering over time in ultrapure water, PBS and acetate buffer.

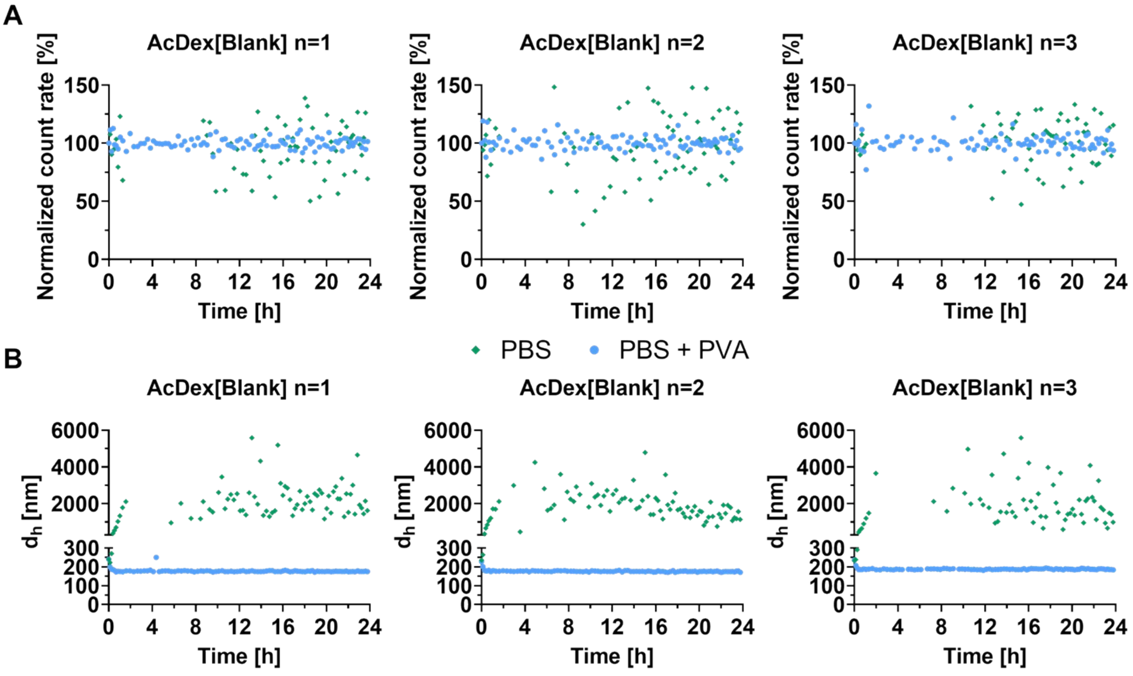

Figure S14: Degradation profile of AcDex[Blank] particles using different media. Degradation was observed by monitoring the (A) count rate and (B) size (z-average value represented as d_h) by dynamic light scattering over time in PBS and PBS with the addition of 0.03 % PVA. n = 3 different batches of manufactured nanoparticles.

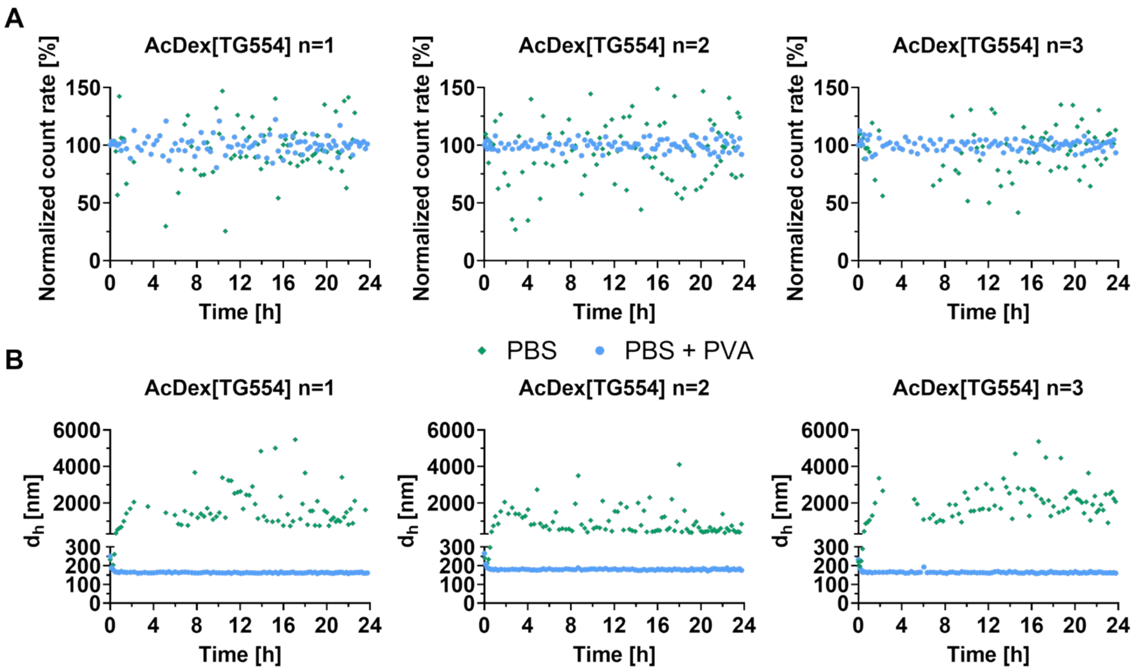

Figure S15: Degradation profile of AcDex[TG554] particles using different media. Degradation was observed by monitoring the (A) count rate and (B) size (z-average value represented as d_h) by dynamic light scattering over time in PBS and PBS with the addition of 0.03 % PVA. n = 3 different batches of manufactured nanoparticles.

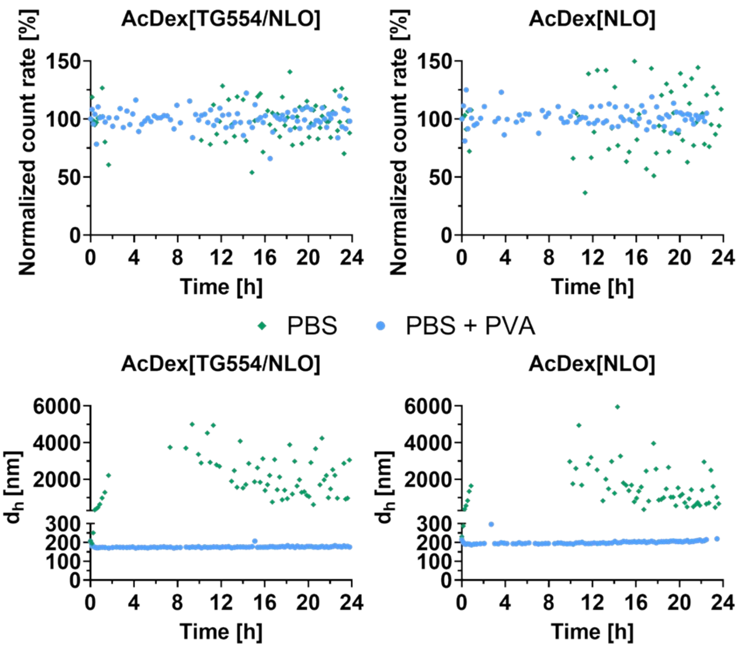

Figure S16: Degradation profile of AcDex[NLO] and AcDex[TG554/NLO] particles using different media. Degradation was observed by monitoring the (A) count rate and (B) size (z-average value represented as d_h) by dynamic light scattering over time in PBS and PBS with the addition of 0.03 % PVA.

4.5 Biosafety and cellular uptake

Table S11: Cytotoxicity of AcDex[TG554] and AcDex[Blank] nanoparticles in M1-MDM at three different concentrations (corresponding to 0.1, 1, and 10 µM of TG554) at 24 h (n = 3 of one formulation batch).

		AcDex [TG554]			AcDex[Blank]		
**Donors**		**Concentration [µg mL^{-1}]**					
	Control	1.64	16.43	164.3	1.64	16.43	164.3
**D1**	100	103.0	95.0	94.4	96.8	95.9	93.9
**D2**	100	95.6	93.7	85.4	93.7	94.4	83.6
**D3**	100	101.8	90.9	95.2	101.3	92.0	96.5

Table S12: MFI values corresponding to the uptake of AcDex[Blank] and AcDex[TG554] in M1-MDM at a concentration corresponding to 1 µM of TG554 (16.43 µg mL^{-1}) at 30 min post-treatment (n = 3 of one formulation batch). MFI = mean fluorescence intensity.

Donors	MFI		
	Control	AcDex[TG554]	AcDex[Blank]
**D1**	5895	26590	22794
**D2**	5317	27462	28213
**D3**	3978	42919	33609

4.6 Bioactivity

Table S13: Summary of individual produced lipid mediators reported per donor as pg/1 x 10^{6} M1-MDM, analysed by UPLC-MS/MS, for n = 3 different batches of manufactured nanoparticles. Related to Figure 5.

Sample	PGE_2	LTB_4	TXB_2
**PBS**	6724.8	3645	14887.4
**AcDex[Blank] n = 1**	6738.2	4858.7	16059.4
**AcDex[Blank] n = 2**	7166.9	5169.8	14818.7
**AcDex[Blank] n = 3**	6552.6	4688.6	13446.1
**AcDex[TG554] n = 1**	1190.3	4400.1	17589.8
**AcDex[TG554] n = 2**	2045.1	5849.3	19462.8
**AcDex[TG554] n = 3**	1733.6	5397.5	18549.4
